# Highly Efficient NIR-Reflective CrAl_2_O_4_‑Based Polymer Microcapsules for Advanced Thermal Management Coatings

**DOI:** 10.1021/acsomega.6c02354

**Published:** 2026-05-15

**Authors:** Jittipat Omsinsombon, Amorn Chaiyasat, Chumphol Busabok, Preeyaporn Chaiyasat

**Affiliations:** † Department of Chemistry, Faculty of Science and Technology, 65171 Rajamangala University of Technology Thanyaburi, Pathum Thani 12110, Thailand; ‡ Advanced Materials Design and Development (AMDD) Research Unit, Faculty of Science and Technology, Rajamangala University of Technology Thanyaburi, Pathum Thani 12110, Thailand; § Expert Centre of Innovative Materials, 318616 Thailand Institute of Scientific and Technological Research (TISTR), Khlong Luang, Pathum Thani 12120, Thailand

## Abstract

Near-infrared (NIR)-reflective materials are of interest for reducing solar heat gain in coated surfaces. In this study, CrAl_2_O_4_ particles were investigated as a novel NIR-reflective pigment. A spinel CrAl_2_O_4_ structure with an average particle size of 1 μm demonstrates high NIR reflectance. The CrAl_2_O_4_ particles were first modified with 3-methacryloxypropyl trimethoxysilane (MPS-CrAl_2_O_4_) to improve their compatibility and dispersion in the polymer matrix. The MPS-CrAl_2_O_4_ particles were encapsulated in polymer microcapsules using UV-initiated microsuspension polymerization. Microcapsules based on methyl methacrylate (MMA), butyl acrylate (BA), and ethylene glycol dimethacrylate (EGDMA) were prepared, and the effects of monomer composition and pigment loading on morphology, thermal behavior, NIR reflectance, and coating performance were examined. A monomer ratio of MMA: BA: EGDMA = 50:40:10 yielded spherical microcapsules with good colloidal stability. The NIR reflectance increased with increasing MPS-CrAl_2_O_4_ loading, reaching 87% at 40 wt %, which was comparable to that of the original CrAl_2_O_4_ particles. Increasing the BA content lowered the glass transition temperature of the microcapsules and promoted coating formation on the glass substrate without an additional binder. Under the specific test conditions used in this work, the microcapsule-coated glass showed a lower interior temperature, with a maximum reduction of about 16 °C, whereas bare glass showed a smaller temperature difference of about 2–3 °C. The results demonstrate that the developed CrAl_2_O_4_ particles exhibited effective NIR reflection and can be integrated into coating systems by polymer encapsulation while retaining their NIR-reflective behavior.

## Graphical abstract

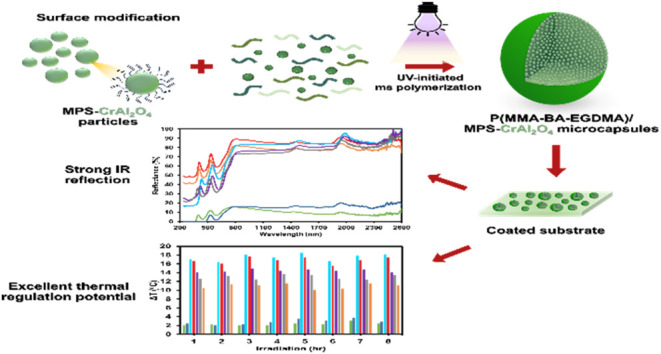

## 1 Introduction

Solar energy represents one of the most abundant and sustainable renewable resources, providing a clean and viable alternative to fossil fuels. However, a significant portion of solar radiation, approximately 53%, falls within the near-infrared (NIR) range (700–2,500 nm),^1,2^ which is a major contributor to heat buildup on the Earth’s surface.^3^ When outdoor surfaces absorb this NIR radiation, it leads to undesirable thermal accumulation, raising the internal temperatures of buildings and subsequently increasing the energy demand for cooling systems.^4^

To moderate this issue, NIR-reflective coatings have been extensively utilized in the construction industry, particularly on building exteriors such as roofs and facades.^5–7^ Many studies on cool-colored pigments are specifically based on the principle of reflecting the NIR portion of sunlight while maintaining the desired visible light. For example, Uemoto et al.^8^ investigated cool-colored paints containing NIR-reflective materials and directly linked their spectral properties to improved thermal performance under solar exposure. These coatings typically incorporate inorganic pigments due to their superior thermal stability, chemical resistance, and long-term durability. However, many conventional pigments rely on toxic heavy metals, including cobalt (Co), cadmium (Cd), and lead (Pb), posing considerable risks to human health and the environment.^9^ As a result, increasing research efforts have been directed toward developing environmentally friendly NIR-reflective pigments composed of nontoxic materials that maintain or improve traditional systems’ performance. Several high-refractive index metal oxides have been explored for their potential in NIR-reflective applications, including titanium dioxide (TiO_2_),^10^ lanthanum–strontium-copper silicates,^11^ bismuth vanadate composites, and cobalt aluminate (CoAl_2_O_4_) spinel pigment.^12^ Among them, spinel-type oxides with a general formula of AB_2_O_4_ have shown particular promise, attributed to their structural stability and excellent NIR reflection.^13^ Chromium aluminate (CrAl_2_O_4_), a spinel-type oxide, has attracted growing interest for its promising NIR-reflective properties. Nevertheless, its potential as the NIR-reflective material has remained largely unexplored. In particular, comprehensive studies investigating its NIR reflection efficiency, dispersion stability within coating systems, and practical performance are still rare. Addressing this critical gap, the present study systematically evaluates the NIR-reflective behavior, dispersion characteristics, and coating performance of CrAl_2_O_4_. To the best of our knowledge, this is among the first detailed investigations to highlight the feasibility of employing CrAl_2_O_4_ as an effective NIR-reflective pigment for thermal management applications.

Cool pigments, materials that exhibit strong absorption in the visible spectrum while reflecting or transmitting NIR radiation, offer an effective means of reducing surface temperatures for energy-saving architectural and surface cooling technology applications by minimizing NIR absorption.^14,15^ NIR-reflective pigments can function independently without requiring a reflective background layer, unlike NIR-transmitting pigments, which typically depend on substrates, such as TiO_2_, to achieve effective thermal regulation. However, many metal oxide pigments, including CrAl_2_O_4_ and other aluminum-based oxides, exhibit poor colloidal stability in aqueous media, particularly in water-based coating formulations.^16,17^ These particles tend to agglomerate, increasing the particle size, reducing the surface area, and diminishing their reflective efficiency. To address these challenges, surface modification of metal oxide pigments is essential to enhance dispersion and maintain long-term stability. Functionalizing the particle surfaces with appropriate chemical groups can significantly improve compatibility with the coating matrix and prevent particle agglomeration.^18,19^ Among the available modification strategies, polymer encapsulation has demonstrated exceptional effectiveness in stabilizing metal oxide pigments.^20,21^ Encapsulating NIR-reflective particles within polymer microcapsules not only enhances the colloidal stability and environmental durability but also improves their distribution within the coating matrix. Furthermore, such an encapsulation facilitates the formation of uniform, self-adhering coatings suitable for direct application onto a wide range of substrates.

Among various encapsulation techniques, suspension polymerization is particularly effective for producing polymer microcapsules encapsulating metal oxide particles.^21,22^ Recognized as one of the most industrially significant methods for polymerization in dispersed systems,^23^ it enables the incorporation of solid particles into polymer matrices through controlled emulsification and polymerization. In this process, monomer droplets containing CrAl_2_O_4_ pigments are stabilized and polymerized to fabricate microcapsules under specific stirring conditions and appropriate initiator systems. Based on its droplet nucleation mechanism, high encapsulation efficiency (EE) is achieved. Moreover, the incorporation of soft polymers with low glass transition temperatures (Tg), such as polybutyl acrylate (PBA), enhances the flexibility and self-coating ability of the resulting microcapsules.^24,25^

UV-initiated polymerization methods provide a rapid and efficient strategy for fabricating polymers, offering fast reaction kinetics, accurate control of position and timing, energy efficiency, and environmental friendliness.^26^ These systems operate at room temperature and represent a cost-effective and sustainable approach to advanced material processing. However, while their use is well-established in synthesis-based processes, applications in dispersion systems remain limited and warrant further investigation. A wide range of photoinitiating systems has been developed for UV-curing processes, with several types of radical photoinitiators commonly used for polymerization in dispersed systems.^27^ These UV initiation strategies enable controlled polymer formation under mild conditions, making them highly suitable for fabricating functional composite microcapsules.

Despite the promising NIR-reflective properties of CrAl_2_O_4_, limited studies have systematically investigated its thermal-reflective performance in coating applications, particularly in relation to particle stability and dispersion within polymer matrices. Moreover, the potential of polymer encapsulation as a strategy to enhance particle stability and coating performance while retaining the intrinsic NIR-reflective behavior of CrAl_2_O_4_ remains largely unexplored. In this study, CrAl_2_O_4_-loaded polymer microcapsules were prepared via UV-initiated microsuspension polymerization to address this gap. The effects of key formulation parameters, including CrAl_2_O_4_ particle size and loading, the MMA/BA monomer ratio, and cross-linker content, were systematically investigated. The primary outcomes considered were microcapsule morphology and stability, NIR reflectance, and temperature-response behavior on coated glass substrates. In addition, the performance of encapsulated CrAl_2_O_4_ particles was directly compared to that of unencapsulated counterparts. Through this approach, this study aims to elucidate the relationship between the formulation design and NIR-reflective coating performance.

## 2 Materials and Methods

### 2.1 Materials

Chromium­(III) oxide (Cr_2_O_3_; Technical grade, Sigma-Aldrich) and aluminum oxide (Al_2_O_3_; Analytical reagent, Sigma-Aldrich) were used as received for the preparation of CrAl_2_O_4_ particles. Oleic acid (OA; Technical grade 90%, Sigma-Aldrich), acetic acid glacial (99.7%, RCI Labscan), 3-(trimethoxysilyl) propyl methacrylate (MPS; Technical grade 98%, Sigma-Aldrich), toluene (99.5% purity, RCI Labscan), and hydroquinone (ReagentPlus, 99% purity, Sigma-Aldrich) were used for the surface modification of CrAl_2_O_4_ particles. Methyl methacrylate (MMA; 99% purity, Sigma-Aldrich) and butyl acrylate (BA; 99% purity, LOBA Chem) monomers and ethylene glycol dimethacrylate (EGDMA; 98% purity, Sigma-Aldrich) as a cross-linked comonomer were purified by passing through a column packed with basic aluminum oxide to remove the contained inhibitors before use. As a photoinitiator, 2-methyl-1-phenyl-2-propanol (MPP; 98% purity, Sigma-Aldrich) was used. Hydroquinone (99%, ReagentPlus, Sigma-Aldrich) was used as an inhibitor. Poly­(vinyl alcohol) (PVA; 89–90% hydrolyzed, average molecular weight 30,000–70,000, Sigma-Aldrich) was used as a stabilizer. Deionized water was used throughout the study.

### 2.2 Preparation of Chromium Aluminate Particles

CrAl_2_O_4_ particles were prepared by a solid-state reaction,^25^ as follows. One mol of Cr_2_O_3_ and 2 mol of Al_2_O_3_ powders were separately dissolved in deionized water (10% solid content). They were then mixed in a ball mill for 24 h at 150 rpm. After that, water was evaporated at 100 °C before the dried powder was calcined at 1,400 °C for 3 h, in an O_2_ atmosphere. Finally, the powder was ground in a ring mill at room temperature. The optimal grinding time was separately investigated at 60 and 240 min, yielding CrAl_2_O_4_ particles.

### 2.3 Surface Modification of Chromium Aluminate Particles

Before preparing polymer microcapsules, surface modification of CrAl_2_O_4_ particles was conducted to increase their hydrophobicity. MPS was dissolved and agitated for 30 min in deionized water, pH 4 (adjusted with acetic acid), before adding CrAl_2_O_4_ particles at different weight ratios of CrAl_2_O_4_: MPS, as shown in Table 1. The mixture was continuously stirred for 2 h at room temperature. Three drops of hydroquinone were then added to the mixture. After that, the dispersion was transferred to a round-bottom flask and stirred at 110 °C for 3 h. The chemical structure of modified MPS-CrAl_2_O_4_ particles was confirmed with the Fourier-transform infrared (FTIR) and X-ray Photoelectron Spectroscopy (XPS).

**Table 1:** Reagent Amounts for the Surface Modification of CrAl_2_O_4_ Particles

		CrAl_2_O_4_/MPS (%w/w)			
chemicals		100:0	50:50	33:67	67:33
CrAl_2_O_4_	g	4.00	2.00	1.33	2.67
MPS	g	0.00	2.00	2.67	1.33
H_2_O	g	100.00	100.00	100.00	100.00

Before microcapsule preparation, a partitioning study of MPS-CrAl_2_O_4_ particles was conducted to evaluate their affinity for the monomer and aqueous phases. MPS-CrAl_2_O_4_ particles (0.07 g) were dispersed in the monomer phase at a concentration of 10 wt % relative to the total monomer content. The monomer phase consisted of MMA and BA at a weight ratio of 50:50. Subsequently, the monomer phase (10 wt % relative to the aqueous phase) was added to water and stirred for 30 min at room temperature. The system was then allowed to stand until a clear monomer-water bilayer was formed.

### 2.4 Preparation of Polymer Microcapsules

Polymer microcapsules containing MPS-CrAl_2_O_4_ particles were prepared by UV-initiated microsuspension polymerization. First, the oil phase was prepared by mixing MMA, BA, or cross-linking monomer and MPP in the desired proportions until a homogeneous solution was obtained. Then, MPS-CrAl_2_O_4_ particles were added to this monomer mixture and dispersed thoroughly to form a uniform oil phase. Separately, an aqueous phase consisting of 1 wt % PVA solution was prepared. The oil phase was then added into the aqueous phase, followed by homogenization at 5,000 rpm for 5 min using a high-speed homogenizer to generate an oil-in-water (O/W) microsuspension. The resulting suspension was transferred to a round-bottom flask, sealed with a silicone rubber septum, and deoxygenated by five vacuum/N_2_ cycles. Polymerization was subsequently carried out under irradiation with a 30 W UV lamp for 1 h at room temperature, under mild stirring. After polymerization, three drops of hydroquinone were added to quench the reaction mixture. The monomer conversion, particle size, and morphology of the obtained polymer microcapsules were studied. The effects of the MMA/BA ratio, the amount of cross-linker, and the content of MPS-CrAl_2_O_4_ particles on the formation and properties of the microcapsules were investigated under the conditions summarized in Table 2.

**Table 2:** Reagent Amounts for the Preparation of Polymer Microcapsules Encapsulating MPS-CrAl_2_O_4_ Particles by Microsuspension Polymerization

		runs										
phases	chemicals		1	2	3	4	5	6	7	8	9	10
oil	MMA	(g)	4.50	4.00	3.50	3.00	2.50	3.50	3.00	1.50	2.00	2.50
	BA	(g)	0.50	1.00	1.50	2.00	2.50	1.50	2.00	2.00	2.00	2.00
	EGDMA	(g)	-	-	-	-	-	-	-	1.50	1.00	0.50
	MPP	(g)	0.40	0.40	0.40	0.40	0.40	0.40	0.40	0.40	0.40	0.40
	MPS-CrAl_2_O_4_	(g)	-	-	-	-	-	0.50	0.50	0.50	0.50	0.50
water	PVA	(g)	0.45	0.45	0.45	0.45	0.45	0.45	0.45	0.45	0.45	0.45
	Water	(g)	44.55	44.55	44.55	44.55	44.55	44.55	44.55	44.55	44.55	44.55

### 2.5 Infrared Ray Reflection and Temperature Control Efficiency

The fabricated polymer microcapsules’ ability to reflect NIR rays was assessed as follows. 5 wt % P­(MMA-BA-EGDMA)/MPS-CrAl_2_O_4_ microcapsules were directly deposited on glass slides as prototype substrates without a binder. After that, it was subjected to water evaporation to create a self-coating layer that adhered to the substrate. By using a UV–vis-NIR spectrophotometer (Shimadzu; UV-3600 Plus; UV/vis/NIR range from 240 to 2,600 nm), it was possible to compare the NIR reflection efficiency of P­(MMA-BA-EGDMA)/MPS-CrAl_2_O_4_ microcapsule films to that of CrAl_2_O_4_ original particles. The NIR reflectance was quantitatively determined using eq 1.^17,18,28^

1 $$R_{\lambda 0\to \lambda 1}=\frac{\int \lambda 1\;\lambda 0\;\mathrm{S\lambda}\cdot \mathrm{r\lambda}\cdot \mathrm{d\lambda}}{\int \lambda 1\;\lambda 0\;\mathrm{S\lambda}\cdot \mathrm{d\lambda}}$$

where R_λ0→λ1_ is defined as the irradiance-weighted average reflectance between wavelength λ_0_ and λ_1_ of its measured spectral reflectance rλ at the wavelength λ.

Sλ is solar spectral irradiance.

The prototype substrates coated with P­(MMA-BA-EGDMA)/MPS-CrAl_2_O_4_ microcapsules were connected to thermocouples positioned above and below the substrates to monitor temperature changes. Each coated substrate was placed 5 cm from a 200 W incandescent lamp (Dahchi). Temperature measurements were recorded for 8 h of light irradiation using four independent coated substrate sheets, with each sample being measured simultaneously. The measured electricity, both above and below the substrates, recorded by the Data Logger connected to the thermocouples, was converted to temperature in degrees Celsius (°C). The temperature differences were then calculated and plotted versus irradiation time.

### 2.6 Durability of Coated Glass Substrate

The accelerated durability and weatherability of the samples were evaluated in accordance with ASTM D4329 ^29^ and ASTM D870,^30^ respectively. Glass substrates coated with P­(MMA-BA-EGDMA) at different MPS-CrAl_2_O_4_ loadings were tested. For each formulation, three independently prepared coated glass specimens (*n* = 3) were examined to confirm experimental reproducibility. In the accelerated UV degradation testing, the samples were exposed to UVA fluorescent lamps with an irradiance of 0.89 W·m^–2^·nm^–1^ at 365 nm under controlled laboratory conditions. The test protocol employed a cyclic exposure procedure, in which each cycle consisted of 12 h of UV irradiation followed by 12 h of water immersion in deionized (DI) water maintained at 30 °C to simulate moisture exposure. After each immersion step, the samples were removed, dried in an oven at 60 °C, and weighed to monitor mass changes. The sample masses were recorded at 24 h intervals to evaluate the durability and degradation behavior of the materials under combined UV radiation and water immersion conditions. This exposure cycle was continuously repeated for a total duration of up to 720 h.

### 2.7 Characterizations

A laser diffraction particle size analyzer (SALD-2300, Shimadzu, Japan) was used to determine the size of CrAl_2_O_4_ and modified-CrAl_2_O_4_ particles. Before analysis, the samples were dispersed in a 1 wt % surfactant aqueous solution and ultrasonicated for 30 min to minimize agglomeration. Each sample was measured three times at room temperature, and the data are presented as particle size distributions. A scanning electron microscope (SEM, JSM-5410, JEOL, Japan) was used to determine the shape of CrAl_2_O_4_ and modified-CrAl_2_O_4_ particles. The dried powder samples were mounted on a nickel SEM stub using conductive adhesive tape and sputter-coated with a thin layer of gold before observation to improve surface conductivity. SEM images were obtained at an accelerating voltage of 15 kV under high-vacuum conditions. An X-ray diffractometer (XRD, Rigaku, SmartLab, Japan) was used to determine the crystalline structure of the CrAl_2_O_4_ and modified-CrAl_2_O_4_ particle powders. Data were collected in the 2θ range of 0°-90° with a step size of 0.0167° and an exposure per step of 27 s. The oxidation states on the surface of CrAl_2_O_4_ (around 5–10 nm) were qualitatively analyzed using X-ray photoelectron spectroscopy (XPS; Kratos AXIS Ultra DLD, U.K.) equipped with a monochromated Al Kα X-ray source. The base pressure in the analysis chamber was maintained at 3 × 10^–4^ Torr. The operating conditions included an emission current of 10 mA and an anode high tension (HT) of 15 kV. A hemispherical analyzer was positioned at a takeoff angle of 180° relative to the sample surface. The chemical structure of MPS-CrAl_2_O_4_ was investigated with an attenuated total reflectance Fourier-transform infrared (ATR-FTIR, NicoletTM iSTM 5, Thermo Fisher Scientific). Before performing an FTIR measurement, the dried MPS-CrAl_2_O_4_ particles were subjected to three ethanol washes to remove any free MPS from the surface in a wavenumber range of 400–4000 cm^–1^. An optical microscope (OM, SK-100EB & SK-100 ET, Seek Inter, Thailand) was obtained using a 40× objective lens and a 10× eyepiece lens, corresponding to a total magnification of 400×, and SEM was used to observe the polymer microcapsules’ inner structure and surface morphology, respectively. The dried polymer microcapsules were mounted on a nickel SEM stub and sputter-coated with a thin Au layer before observation. The incorporation and distribution of CrAl_2_O_4_ particles in the polymer microcapsules were investigated using energy-dispersive X-ray spectroscopy (EDS) attached to a scanning electron microscope (SEM, JSM-5410, JEOL, Japan). Both SEM and EDS analyses were carried out at an accelerating voltage of 5 kV under vacuum conditions. Percent monomer conversion was determined by gravimetry using three replicate measurements, and the average values were reported. Suspension samples (about 1.0 g) were collected from the reactor and placed directly into an aluminum cup, where they were precisely weighed. Before the free liquid evaporated in an oven at 80 °C, a few drops of hydroquinone solution (1 wt %) were added to the suspension. A constant weight of the dried polymer was obtained after drying the sample. Monomer conversion was determined by comparing the weight of the dried polymer with that of the original monomer. The Tg values of polymer microcapsules at various monomer ratios were measured using a differential scanning calorimeter (DSC, DSC 4000, PerkinElmer) at a scanning rate of 10 °C/min under a nitrogen atmosphere with a 20 mL/min flow rate. The existing CrAl_2_O_4_ content or experimental loading (LE) in polymer microcapsules was determined using a thermogravimetric analyzer (TGA; TGA 4000, PerkinElmer). The measurements were carried out over a temperature range of 30–800 °C at a heating rate of 10 °C min^–1^ under a nitrogen atmosphere with a 20 mL/min flow rate.

## 3 Results and Discussion

### 3.1 Preparation of CrAl_2_O_4_ Particles

A simple solid-state reaction was used to create the CrAl_2_O_4_ particles. The green CrAl_2_O_4_ powder (Figure 1a) was produced after water evaporation and calcination. In principle, when a light beam strikes a sample that is in powder form, reflection, transmission, and absorption may happen. If the sample is sufficiently optically thick, the transmitted light is insignificant. Specular and diffuse reflections are the two main forms of reflection. For highly absorbing materials and optically flat surfaces, specular reflection is important. As the incoming radiation enters the sample and reflects off the grain boundaries of the particles, diffuse reflection occurs. Particle size affects diffusion reflection in such cases. The number of reflections at the grain boundaries rises as particle size decreases. Consequently, the depth of incoming light penetration reduces, which causes a drop in absorption and an increase in reflection. The amount of light absorbed then drops, while the amount of reflected light rises.^9^ To be used as an NIR-reflective material, its crystalline structure significantly affects its performance. Then, the crystalline structure of the synthesized CrAl_2_O_4_ particles was determined with XRD. The XRD pattern of CrAl_2_O_4_ is presented in Figure 1b, along with the original patterns of Cr_2_O_3_ and Al_2_O_3_ for comparison. CrAl_2_O_4_ exhibits diffraction peaks located at approximately 2θ = 25.4°, 36.9°, 45.2°, 55.7°, 59.3°, 65.1°, and 75.4°, corresponding to the (220), (311), (400), (511), (440), (622), and (444) planes of the cubic spinel structure (JCPDS No. 33–0458, space group Fd^3^m).^31^ The most intense peak at around 36.9° can be indexed to the (311) plane, which is characteristic of the spinel-type CrAl_2_O_4_ phase.^32^ No additional peaks corresponding to Cr_2_O_3_ (corundum-type, PDF No. 38–1479) or Al_2_O_3_ (γ- or α-phase) are detected,^33^ indicating that the synthesized samples are composed of a spinel structure with high purity. The spinel structure demonstrates excellent NIR reflection owing to its thermodynamically stable cubic lattice, wide band gap, and the absence of NIR-active vibrational modes. These characteristics effectively suppress NIR absorption and enhance reflection across the NIR region. As a dielectric ceramic, spinel minimizes interactions with electromagnetic radiation, while its microstructural features contribute to increased light scattering, further improving NIR reflectivity.^34^

**Figure 1:**
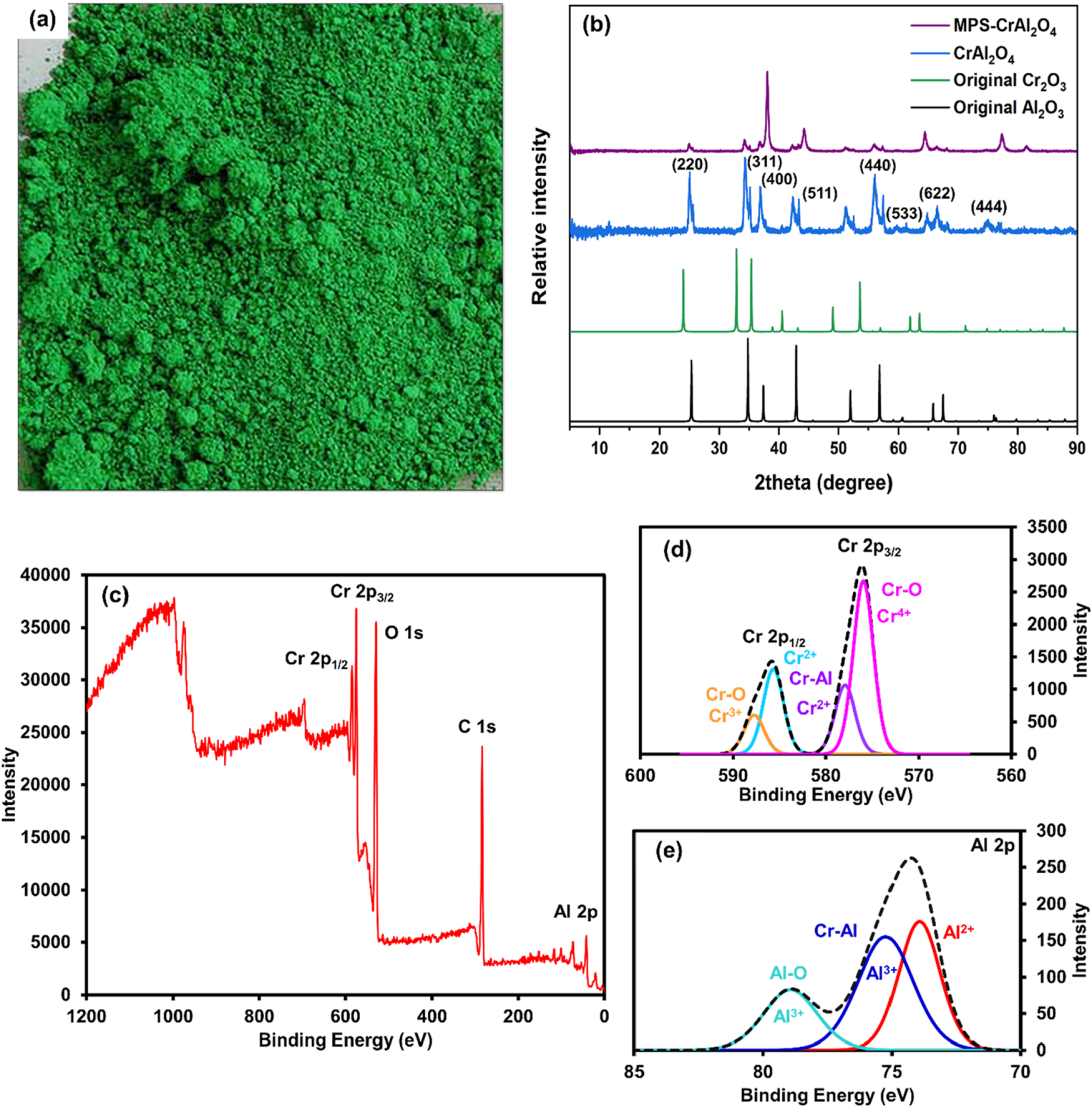
Photo (a), XRD patterns (b) of original Cr_2_O_3_, Al_2_O_3_, CrAl_2_O_4_, and MPS-CrAl_2_O_4_ particles, and XPS wide-scan spectrum (c) and high-resolution XPS Cr 2p (d) and Al 2p (e) spectra of CrAl_2_O_4_ particles.

XPS was used to classify the elements and states of the top surface CrAl_2_O_4_ (approximately 5–10 nm), as shown in Figure 1c–e. Cr 2p and Al 2p peaks were seen in the CrAl_2_O_4_ survey scan XPS spectrum, indicating that Cr and Al elements were present in the compound. The peaks at 585.7 and 576.0 eV in Figure 1d were attributed to Cr 2p_1/2_ and Cr 2p_3/2_, respectively.^35^ The two binding energies of the Cr 2p_3/2_ spectra, 576.0 and 578.0 eV, correspond to the Cr–O and Cr–Al groups, respectively, corresponding to the +4 and +2 oxidation states.^36^ The Cr 2p_1/2_ oxidation state of Cr^2+^ and Cr^3+^ was said to be responsible for the peaks at 586 and 588 eV, respectively.^37,38^ Figure 1e shows the Al 2p peaks at 74.0, 75.2, and 79.0 eV for Al^2+^, Al^3+^, and Al^3+^, respectively, corresponding to the Al–O and Cr–Al groups.^36,39,40^ These findings indicate that the detected chromium and aluminum species were consistent with the oxidation state of chromium and aluminum in the CrAl_2_O_4_ structure, which was successfully prepared. The coexistence of the Cr^4+^ and Al^2+^-like components observed in the XPS spectra indicates a charge-compensation mechanism driven by oxygen-vacancy formation within the CrAl_2_O_4_ spinel lattice. The partial oxidation of Cr^3+^ to Cr^4+^ at the surface is balanced by a corresponding partial reduction of neighboring Al^3+^ to Al^2+^-like suboxide species. This redox coupling (Cr^3+^ → Cr^4+^ and Al^3+^ → Al^2+^-like) maintains local charge neutrality and is consistent with oxygen-deficient environments typically formed during high-temperature calcination or surface oxidation. Similar defect-assisted valence adjustments have been reported for other spinel-type MAl_2_O_4_ oxides.^41^

After the obtained CrAl_2_O_4_ was processed in the ring mill, SEM images show micrometer-sized CrAl_2_O_4_ particles (Figure 2a,b). By dispersing particles in water (1 wt %) to ensure proper dispersion throughout the measurement, laser diffraction particle size analyzers assessed the average CrAl_2_O_4_ particle sizes ± SD of 9.78 ± 0.49 and 1.03 ± 0.43 μm for 60 and 240 min of grinding, respectively, based on three repeated measurements. However, large bimodal particle size distribution curves (Figure 2a′,b′), which reflect the two primary particle sizes, were seen, particularly in the case of 60 min grinding. Because the particles were prepared by solid-state reaction, after calcination, the obtained product formed as a solid mass. It was subsequently ground into smaller particles using a ring mill, i.e., a mechanical size-reduction process in which the final particle size is strongly influenced by the grinding conditions rather than being inherently uniform. Recent studies have shown that mechanical grinding can markedly alter particle size distribution depending on processing parameters, and that mechanically ground powders may exhibit broad or multimodal distributions.^42,43^ In our work, therefore, the grinding time was optimized to obtain particles of a suitable size for effective NIR reflection.

**Figure 2:**
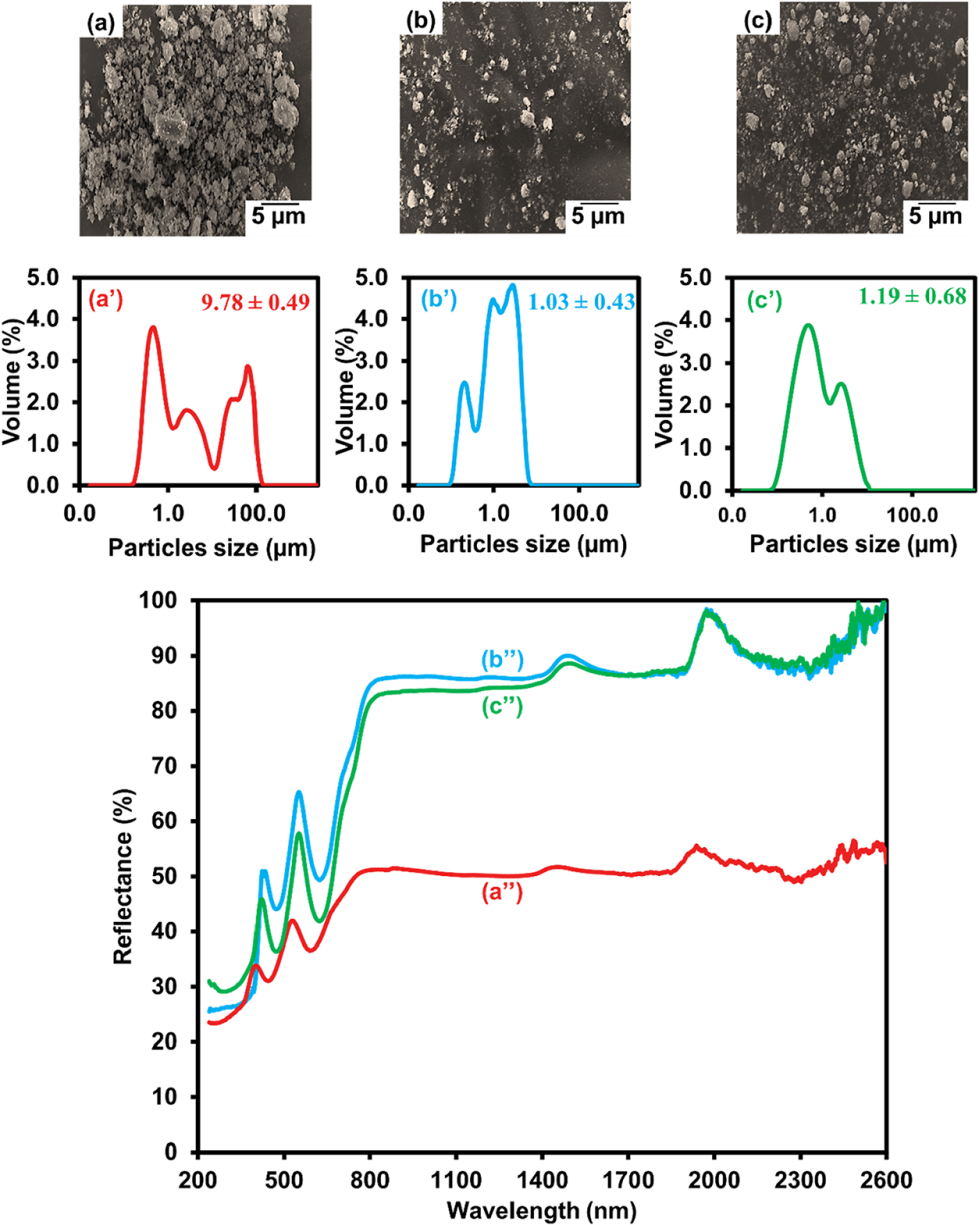
SEM micrographs (a, b, c), size distribution curves (a′, b′, c′) [Data are presented as mean ± standard deviation (*n* = 3)], and IR reflection efficiency (a″, b″, c″) of CrAl_2_O_4_ particles ground in a ring mill at various times (min): 60 (a, a′ and a″); 240 (b, b′ and b″); and (MPS-CrAl_2_O_4_) (c, c′ and c″) ground in a ring mill at 240 min.

Given the crucial impact that particle size plays in solar reflectance, the relationship between particle size and solar wavelength is one of the most important selection criteria for particle size.^44^ According to Fresnel’s rule, the particle size of good NIR-reflective materials must be at least half (d = λ/2) the wavelength of the NIR rays (700–2,500 nm).^44^ For particles to reflect 700–1,100 nm short-wave NIR, their appropriate size must be between 350 and 550 nm. Similarly, reflecting 1,100–2,500 nm long-wave NIR requires a size distribution of about 550–1,250 nm.^44^ Highly effective NIR reflection might be possible with the correct particle size. In the case of 240 min of grinding (Figure 2a′), the weight percentage of smaller particles (0.09–1.25 μm) was higher (56.1%) than that in the case of 60 min of grinding (41.2%) (Figure 2b′). Therefore, the particles ground for 240 min were expected to exhibit increased NIR reflection effectiveness. The NIR reflection performance was determined for both particle sizes to prove this assumption. The findings show that the produced CrAl_2_O_4_ particles have excellent NIR reflection properties. The NIR reflection effectiveness of the smaller-sized particles was much greater than that of the bigger ones, as predicted, as shown in Figure 2, along with both short- and long-wave NIR. The smaller-sized ones had an irradiance-weighted average reflectance value (R) of 89.70% (Figure 2a″), which was almost twice as high as the bigger ones’ (51.13%) value (Figure 2b″). Moreover, this reflectance value is also higher than that of several reported NIR-reflective materials, as in Table S1.^10–12,45–53^ Although TiO_2_-based systems demonstrate relatively good NIR reflection performance, CrAl_2_O_4_ consistently shows higher reflection efficiency. In contrast, other materials such as lanthanum–strontium-copper silicates and CoAl_2_O_4_ display noticeably lower NIR reflection. These results clearly demonstrate that CrAl_2_O_4_ is a highly effective reflective material.

The high NIR reflection of CrAl_2_O_4_ originates from its spinel electronic structure and lattice dynamics.^54–56^ CrAl_2_O_4_ is a wide-band gap oxide, so NIR photon energies are insufficient to induce interband electronic transitions, resulting in intrinsically low NIR absorption.^56,57^ The crystal-field d–d transitions of Cr^3+^ mainly occur in the visible region and are responsible for color, while their contribution to NIR absorption is minimal in the absence of defect- or impurity-induced midgap states.^56^ In addition, IR-active phonon modes in spinel lattices predominantly absorb in the mid- to far-IR region rather than in the NIR, leaving the NIR window largely free from lattice absorption.^58,59^ Under these conditions of low intrinsic absorption, diffuse light scattering from CrAl_2_O_4_ particles—especially when particle sizes are half to NIR wavelengths—further enhances the measured NIR reflection.^54,57,60^ However, the present CrAl_2_O_4_ particles may still absorb a portion of the UV–visible light while simultaneously reflecting a meaningful fraction of NIR radiation. Similar discussions have appeared in previous studies^61,62^ where dark or colored materials were shown to absorb visible light, yet still provide cooling benefits when their NIR reflectance was enhanced.^63,64^ Accordingly, the CrAl_2_O_4_ particles first proposed in this work can be used as a novel IR reflective material with excellent NIR reflection efficiency, particularly for applications requiring high solar reflectivity, such as passive cooling and thermal management technologies.

As previously mentioned, the particle size strongly affects the ability to reflect. A good distribution in various matrices to maintain their particle size is required to effectively use the CrAl_2_O_4_ particles for NIR reflection and temperature control. Furthermore, the durable coating on various substrates enhances their application. Modifying such particles with polymers in the form of capsules or composite particles would be more advantageous than using them directly. Then, polymer microcapsules containing CrA_2_O_4_ particles were created in this work.

### 3.2 Surface Modification of Chromium Aluminate Particles

For successful encapsulation of CrAl_2_O_4_ particles via suspension polymerization, it is essential that the particles are uniformly dispersed within the monomer droplets or oil phase. However, metal oxide particles typically exhibit a strong affinity for aqueous media due to their inherently hydrophilic nature,^65^ which presents a challenge for achieving uniform dispersion in the oil phase. Then, surface modification is required. Modifying their surface with a silane group to increase their hydrophobicity and double bond is a straightforward alternative method.^66^ The efficacy of silane modifiers in forming a strong chemical connection between organic and inorganic materials is their crucial quality. The silane modifiers (X­(CH_2_)*_n_* SiR_3_) contain two functional groups. The organic functional group (X) was selected for its compatibility or reactivity with organic materials. Hydrolyzable groups (R), such as methoxy, ethoxy, and others, are intermediates in the production of silanol groups (Si–OH) for attaching to inorganic or nanoparticle (NP) surfaces.^67^ Among them, 3-(trimethoxysilyl) propyl methacrylate (MPS) is one of the important silanes that is widely used as a functionalization agent to promote interfacial interactions between metal oxide nanoparticles and a polymeric matrix, such as dental acrylic resins. The silanization mechanism occurs by M–O–Si (M = metal) bonds between metal oxide nanoparticles and silane coupling agents.^68^ Moreover, the presence of a double bond in the molecule enables further radical polymerization with other vinyl monomers. It promotes effective immobilization of CrAl_2_O_4_ particles within the polymer matrix and enhances their dispersion, thereby preventing particle agglomeration. Consequently, the NIR reflection performance is maintained without a significant reduction.

The most common technique for surface modification of NPs with silanes is hydrolysis in aqueous solvents such as water or a solution of water and a polar solvent (ethanol, propanol, or acetone).^69,70^ Generally, the silane coupling agent’s alkoxy groups (-OR; -OCH_3_, -OC_2_H_5_) hydrolyze to the Si–OH group by the water in the solution. Then, the condensation reaction occurs between Si–OH and −OH groups on the NP’s surface. The hydrolysis of silanes can be done in an aqueous medium with various pH levels (acidic, alkaline, and neutral media). In an alkaline condition, the nucleophilic hydroxyl group attacks the silicon atom in alkoxysilane, and it causes the release of an alcohol (ROH) molecule. The kinetic studies revealed that the hydrolysis rate is slow under neutral conditions. In an acidic condition, the alkoxide group is first protonated. After that, the condensation reaction occurs, and a molecule of ROH is released.^71^ The rate of the hydrolysis process increases under acidic pH, and the resultant silanols are very stable.^71^ Therefore, the modification of CrAl_2_O_4_ particles with MPS in an acidic aqueous solution is used in this work to form MPS-CrAl_2_O_4_ particles.

The XRD pattern of MPS-CrAl_2_O_4_ is shown in Figure 1b, together with those of pristine CrAl_2_O_4_ and the reference patterns of Cr_2_O_3_ and Al_2_O_3_ for comparison. The diffraction peaks of MPS-CrAl_2_O_4_ are consistent with those of the original CrAl_2_O_4_ particles, indicating that the surface modification with MPS does not alter the crystalline structure of CrAl_2_O_4_. Then, the surface functionalization occurred without disrupting the spinel lattice framework.^72^ These results confirm that both CrAl_2_O_4_ and MPS-CrAl_2_O_4_ possess a well-crystallized cubic spinel structure, with Cr^2+^ cations occupying the tetrahedral (A) sites and Al^3+^ cations located at the octahedral (B) sites within the spinel lattice.^72^ The enhanced diffraction peak at 2θ ≈ 38.06° is likely a result of the modification process with MPS, which is an amorphous compound. The change in the peak could indicate a shift in the intensity or a slight modification in the crystallographic orientation due to the surface functionalization by MPS, without altering the overall spinel phase.^73^ Since MPS itself is amorphous, the observed changes at this peak are most likely related to the improved dispersion of the particles or the exposure of specific crystallographic planes due to the surface modification, rather than the formation of a new crystalline phase. Surface functionalization can induce changes in peak intensities or subtle shifts due to interactions between the MPS molecules and the particle surfaces, which affect the crystallite sizes or the preferred orientation of the crystals. This effect is commonly observed in XRD patterns of surface-modified materials, where surface interactions alter the X-ray scattering intensity without causing significant phase changes.

The morphology and average particle size of MPS-CrAl_2_O_4_ were investigated by using SEM and laser diffraction, respectively, in comparison with CrAl_2_O_4_. As shown in Figure 2, surface modification with MPS does not induce noticeable changes in particle morphology or average particle size, with all values remaining comparable within the experimental error. Consistently, the reflectance spectra show similar trends. This indicates that the silane modification occurs predominantly at the particle surface without altering the intrinsic crystal structure or inducing particle growth or fragmentation. Instead, the primary role of MPS is to tailor the surface chemistry of CrAl_2_O_4_ particles, improving dispersion and suppressing agglomeration while preserving the original particle morphology and size.

The FTIR spectra of MPS-CrAl_2_O_4_ particles compared with pristine CrAl_2_O_4_ particles and MPS are shown in Figure 3a–c. Two prominent peaks of MPS were observed at 2,854 and 2,923 cm^–1^, corresponding to the symmetric and asymmetric −CH_2_ stretching, respectively, whereas the carbonyl peak of the ester group was observed at 1,710 cm^–1^.^74^ All of them were observed in the spectrum of MPS-CrAl_2_O_4_, whereas characteristic peaks of CrAl_2_O_4_ at 579 cm^–1^ related to Cr–O and 722 cm^–1^ related to the Al–O bond were also found. These indicated that the CrAl_2_O_4_ particles were successfully coated with MPS.

**Figure 3:**
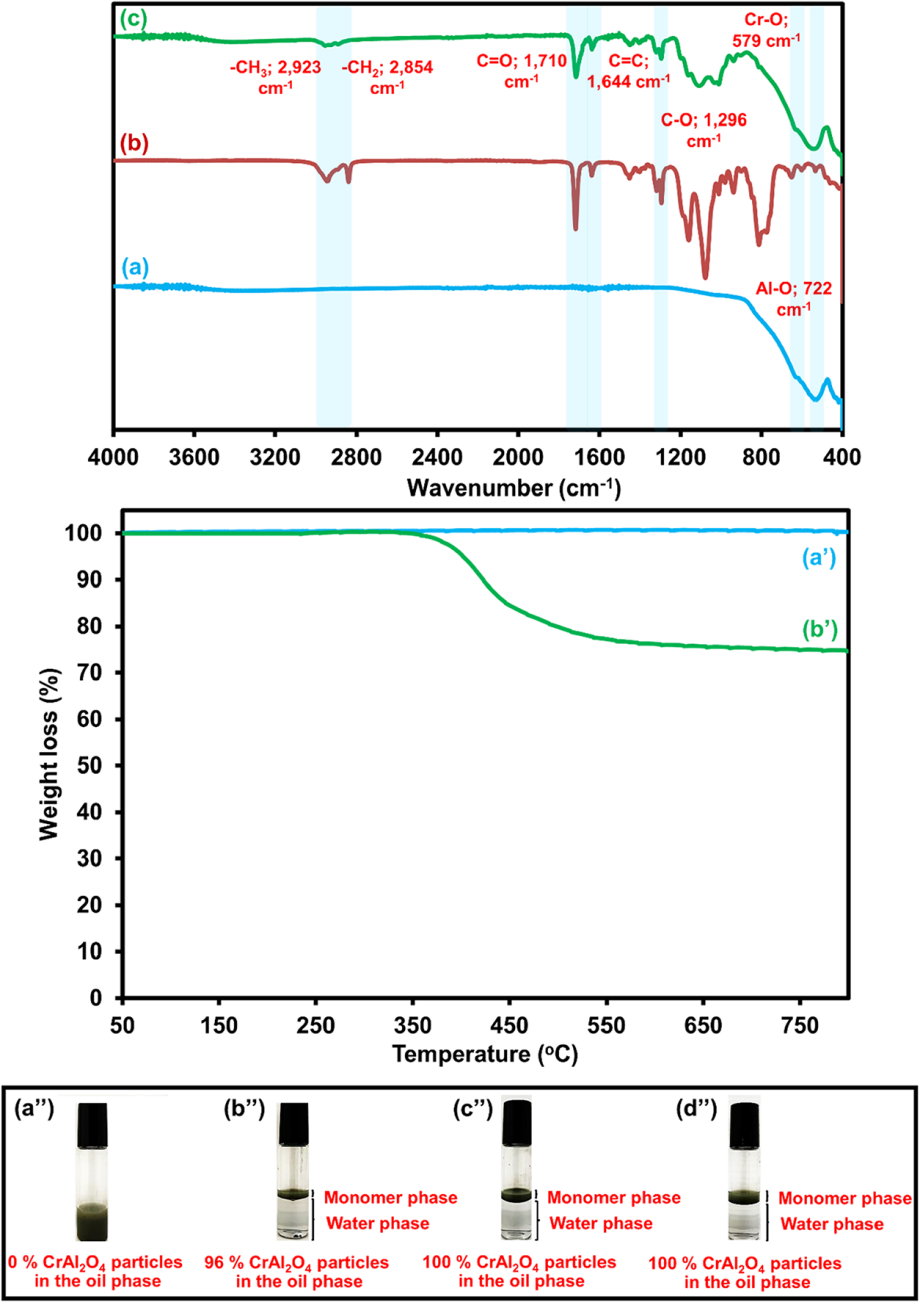
FTIR spectra (a–c) of CrAl_2_O_4_ (a), MPS (b), and MPS-CrAl_2_O_4_ particles (c), TGA cureves (a′, b′) of CrAl_2_O_4_ (a′) and MPS-CrAl_2_O_4_ (b′) particles and binary phase photos (a″–d″) of partitioning study of MPS-CrAl_2_O_4_ particles at various CrAl_2_O_4_: MPS ratios (%w/w): 100:0 (a″); 67:33 (b″); 50:50 (c″); and 33:67 (d″)

The MPS-CrAl_2_O_4_ consists of an inorganic CrAl_2_O_4_ core coated with organic MPS molecules. MPS decomposes at relatively low temperatures, whereas CrAl_2_O_4_ remains thermally stable up to temperatures above 800 °C. Therefore, the amount of MPS can be estimated based on the thermal decomposition behavior. The actual amount of MPS grafted onto the CrAl_2_O_4_ surface was then quantified using TGA. 10 mg of each sample was measured under N_2_ atmosphere. TGA results of pristine CrAl_2_O_4_ and MPS-CrAl_2_O_4_ are displayed in Figure 3a′,b′. The pristine CrAl_2_O_4_ (sky blue line) showed almost no weight loss up to 800 °C, confirming its excellent thermal stability and the absence of any organic residues. In contrast, the MPS-CrAl_2_O_4_ sample (green line) exhibits a significant weight loss of approximately 24.3 wt % between 200 and 600 °C, which can be attributed to the thermal degradation of the organic components of grafted MPS molecules anchored onto the CrAl_2_O_4_ surface. This considerable mass loss clearly demonstrates that a substantial amount of MPS was successfully introduced onto the CrAl_2_O_4_ surface. The modification, therefore, produced an organic–inorganic hybrid structure in which the silane molecules are chemically bonded to the hydroxylated surface of the spinel particles. The high MPS content further supports the effective surface functionalization of CrAl_2_O_4_.

The wettability behavior of the particles was measured by contact angle, which is a common method for evaluating surface hydrophilicity/hydrophobicity. The results showed that the unmodified CrAl_2_O_4_ particles were hydrophilic, while the MPS-CrAl_2_O_4_ particles became highly hydrophobic, confirming successful surface modification, as shown in Figure S1. To verify the effectiveness of the surface modification, monomer phase partitionings of the original CrAl_2_O_4_ and MPS-CrAl_2_O_4_ particles were conducted. As shown in Figure 3a″–d″, when the suspension was stirred and left to form a layer, the distribution of the MPS-CrAl_2_O_4_ particles in the monomer phase increased with MPS concentration. Without MPS, most original CrAl_2_O_4_ particles gathered at the water phase’s bottom (Figure 3a″). In contrast, most particles floated to the upper monomer layer when the CrAl_2_O_4_: MPS was raised from 67:33, 50:50, and 33:67 (Figure 3b″–d″). At the lowest ratio of 50:50, all CrAl_2_O_4_ particles were entirely distributed. Thus, an MPS-CrAl_2_O_4_ particle with such a ratio was chosen for further investigation.

### 3.3 Preparation of Polymer Microcapsules

Among various polymerization techniques in dispersed systems, suspension polymerization is one of the most common methods in industrial practice.^23,75^ Initially, basic mechanical stirring was used to create monomer droplet suspensions. The droplet size distribution, which is typically broad and ranges between 50 μm and 1 mm, is controlled by the interaction between droplet coalescence and breakup. The initiator, dissolved in the dispersion phase, may be either hydrophilic or hydrophobic, depending on whether the suspension is oil-in-water (O/W) or water-in-oil (W/O). A stabilizer, most frequently a water-soluble polymer, is often added to avoid macroscopic phase separation. The polymer microparticles usually have a size distribution close to that of the droplets from which they are generated. While particles are generated directly from monomer droplets, suspension polymerization is conceptually similar to miniemulsion polymerization, even though the kinetics in the latter case are drastically different because of severe compartmentalization. Increasing the stabilizer amount and stirring rate may generate smaller particles via microsuspension polymerization. Microsuspension polymerization proceeds via a droplet nucleation mechanism in which monomer droplets dispersed in an aqueous phase function as individual microreactors. Polymerization takes place within these droplets, leading to the formation of well-defined spherical polymer particles. This mechanism enables a high EE, making the technique particularly advantageous for preparing polymer microcapsules. The core substance is well-dissolved or -dispersed in the monomer droplets. After the polymerization starts, polymer chains are produced in the droplets until they reach their critical chain length, and then, phase separation occurs. The formed polymer chains move to the monomer droplet interface and create the polymer microcapsule shell, thereby encapsulating the core substance. Because of its high EE, microsuspension polymerization is applied for microcapsule fabrication in various applications such as pharmaceutical, agricultural, and controlled release.

Photopolymerization is a light-triggered polymerization process that has attracted increasing attention due to its fast reaction kinetics, accurate control of position and timing, and energy-efficient operation.^26^ Its low-temperature operation and minimal chemical residue make it particularly advantageous for heat-sensitive and biocompatible systems. In this process, photoinitiators absorb light, typically in the UV or visible range, and subsequently generate reactive species such as free radicals or cations, which initiate the polymerization of monomers. In this work, MPP is employed as an effective hydrogen donor in type II photoinitiating systems under UV irradiation. Upon photoactivation, it generates a resonance-stabilized benzylic radical at the tertiary carbon center, efficiently initiating free radical polymerization. Owing to its high photoreactivity, structural stability, and broad compatibility with various monomer systems, MPP represents a promising co-initiator for UV-curable coatings and advanced functional polymer applications.^27,76^ Photopolymerization has been extensively applied to coatings, adhesives, additive manufacturing (3D printing), and biomedical materials. However, there are a few examples of photopolymerization in a dispersed system. Then, in this work, photoinitiated microsuspension polymerization is studied. Several parameters were considered to develop high-performance MPS-CrAl_2_O_4_ polymer microcapsules for solar-heat reflection.

#### 3.3.1 Ratio of MMA/BA

MMA was selected as the main structural monomer because methacrylate-based shells, especially PMMA-rich systems, are widely recognized for their good chemical stability and mechanical strength. In this work, MMA was incorporated to form a relatively rigid and stable polymer matrix, which helps preserve particle integrity and enhances the retention of the encapsulated component. This agrees with previous studies^25,26^ reporting that PMMA-based shell materials have been successfully used in encapsulation systems and can provide high encapsulation efficiency in MMA-containing microcapsules. PBA is a sticky polymer that readily forms a polymer film at ambient temperature because of its low Tg.^24,25^ As a result, BA is employed in this work as a comonomer to produce copolymer particles with self-coating properties. The impact of the MMA: BA ratio on the P­(MMA-BA) shell′s property was examined. After polymerization, white milky suspensions of spherical micrometer-sized polymer particles (Figure 4 (a–d and a′–d′)) were seen at various MMA: BA ratios. Coagulation-free P­(MMA-BA) particle suspensions were generated except at a 50:50 ratio. The particles become increasingly coagglomerated over time, influencing the determination of the conversion percentage.

**Figure 4:**
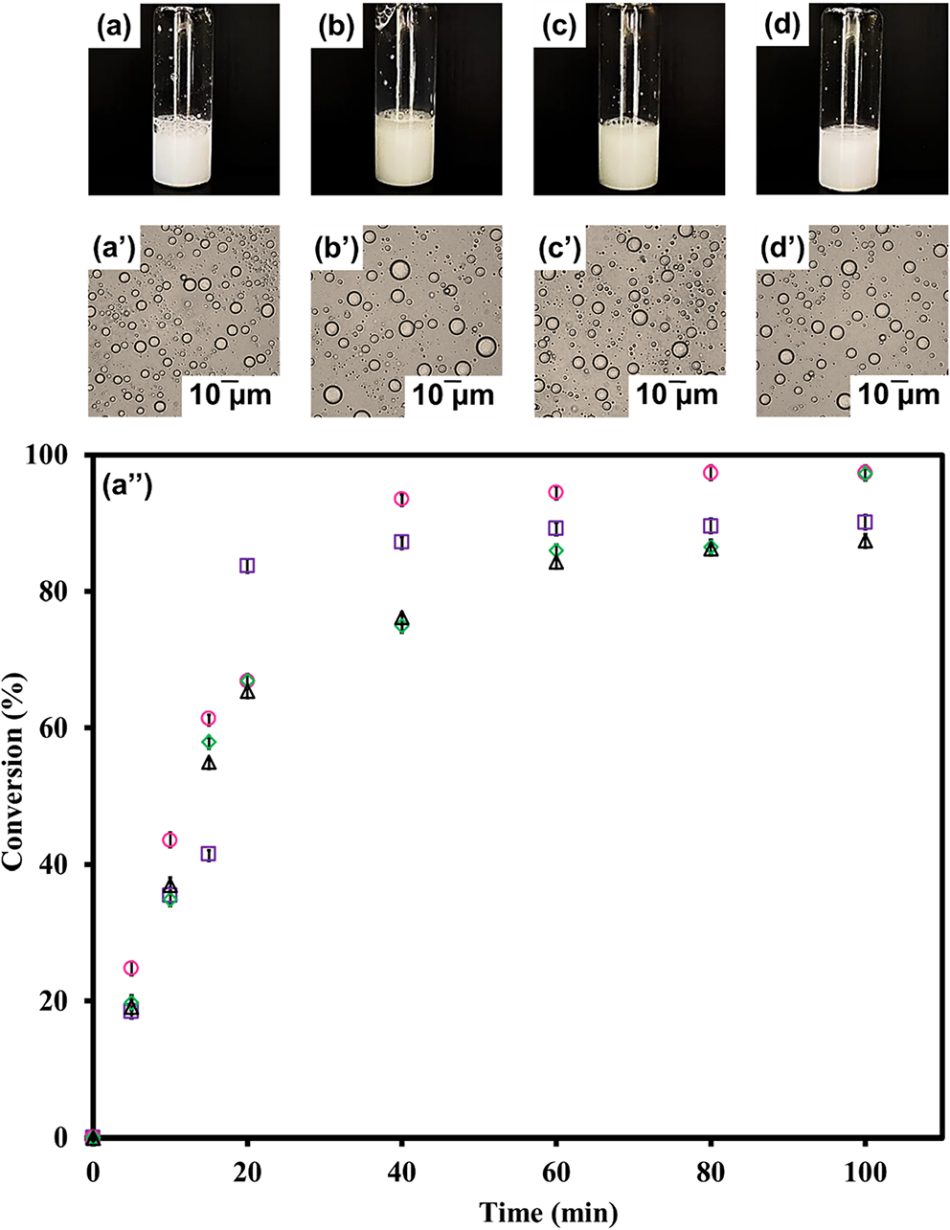
Suspension photos (a–d), optical micrographs (a′–d′), and percent conversions (a″) of P­(MMA-BA) particles prepared by suspension polymerization at various ratios of MMA: BA (%w/w): 90:10 (a, a′ and purple box); 80:20 (b, b′ and pink circle); 70:30 (c, c′ and green diamond); and 60:40 (d, d′ and black trianlge) [Data are presented as mean ± standard deviation (*n* = 3)].

The experimental results revealed that the percentage of conversion increased with increasing polymerization time (Figure 4). Polymerization occurs quickly in the early stages, particularly within the first 20 min. Until 60 min, the percentage conversion was greater than 85% in 4 conditions and remained relatively stable thereafter. It differs significantly from the synthesis with a thermal initiator, which takes a long time to synthesize and requires high temperatures.^22,25^ Because polymerization proceeds easily at ambient temperature with a short polymerization time, UV photoinitiation is attractive for the manufacture of polymer microcapsules in this study. A polymerization time of 60 min was selected as the optimum condition.

The thermal properties of polymer particles are determined to evaluate their film-forming ability. The copolymer’s Tg increased with the lowering of the BA content, as shown in Table 3 and Figure S3. Given the film-forming and self-coating capabilities, high BA at MMA/BA ratios of 60:40 and 70:30, providing lower Tg values than room temperature, were chosen for preparing polymer microcapsule encapsulated CrAl_2_O_4_.

**Table 3:** Characterization Information of P­(MMA-BA) Particles Prepared by Microsuspension Polymerization at Various MMA/BA Ratios^a^

MMA/BA	conversion (%) (±SD*)	Tg (^O^C)
50:50	-(coagulation)	-
60:40	84.36 (±0.39)	3.3
70:30	85.99 (±0.78)	8.6
80:20	94.53 (±0.94)	32.3
90:10	89.26 (±0.55)	49.0

a *n* = 3.

Using such ratios, the encapsulation efficiency of MPS-CrAl_2_O_4_ particles in the P­(MMA-BA) shell was investigated. After polymerization, noncoagulating milky green P­(MMA-BA)/MPS-CrAl_2_O_4_ microcapsule suspensions were generated (Figure 5a,b). Most of the polymer microcapsules precipitated after centrifugation at 3,000 rpm due to their high total densities, whereas the resulting supernatants were fairly translucent. A few free polymer particles (8–10 wt %) with an average size of 200 nm were discovered in aqueous supernatants.

**Figure 5:**
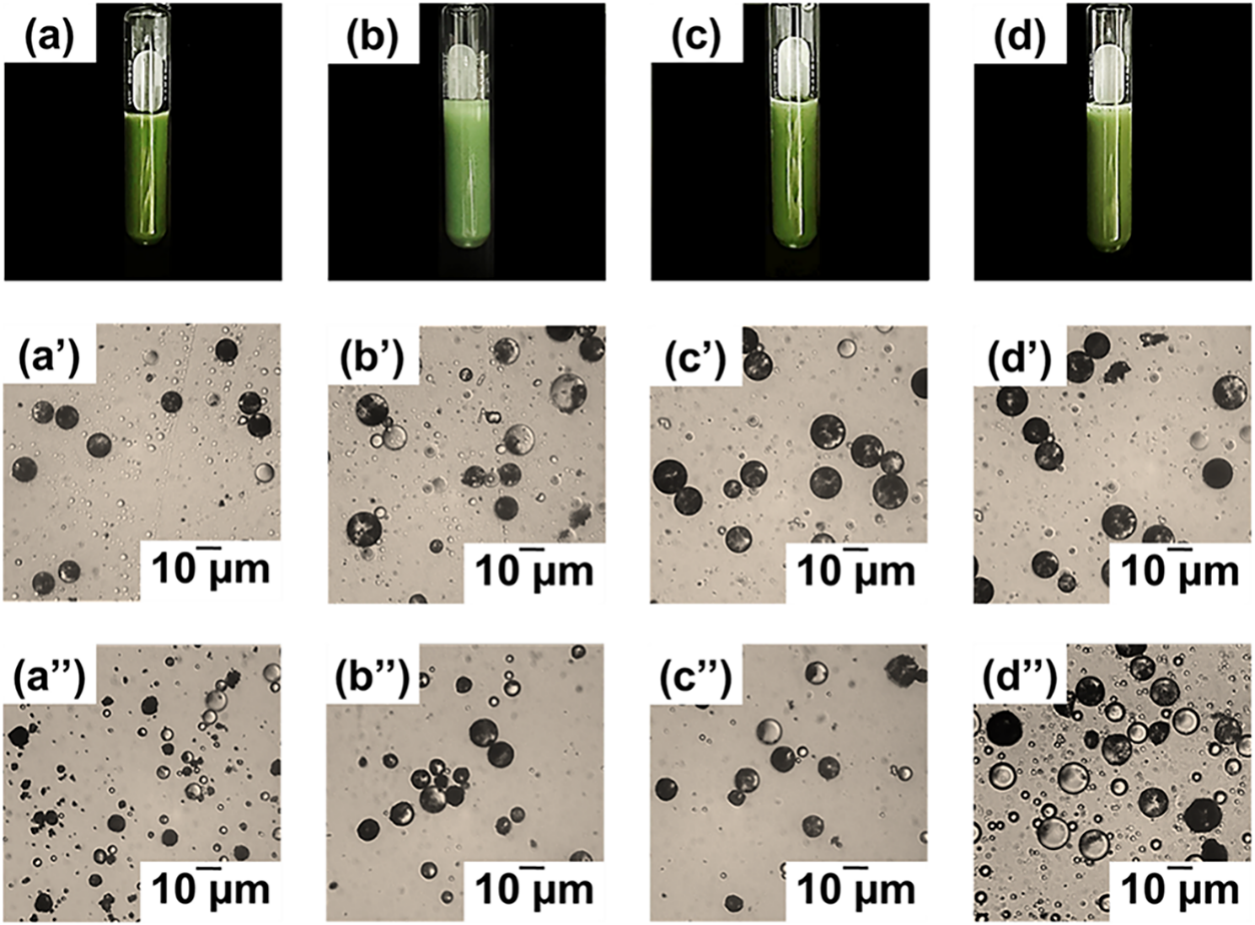
Suspension photos (a–d) and optical micrographs of P­(MMA-BA) microcapsules encapsulating MPS-CrAl_2_O_4_ (a, b) and o-CrAl_2_O_4_ particles (c, d) before (a′–d′) and after (a″–d″) polymerization using various ratios of MMA/BA (%w/w): 60:40 (a, a′, a″, c, c′ and c″) and 70:30 (b, b′, b″ d, d′ and d″)

OM observations of monomer droplets and polymer microcapsules at different MMA: BA ratios are illustrated in Figure 5. By microsuspension polymerization, well-distributed monomer droplets (Figure 5a′,b′) and polymer microcapsules (Figure 5a″,b″) were spherical and ranged in average size from 11 to 12 μm (Figure S2). The high mechanical shear rate-generated monomer droplets and polymer microcapsules are well-known for having a wide range of particle sizes. Based on the droplet nucleation mechanism, the size of microcapsules is similar to monomer droplets, determined by the shear rate and an efficient stabilizer. The observation of numerous MPS-CrAl_2_O_4_ particles as dark regions in the monomer droplets and polymer microcapsules indicated effective encapsulation without any deformation.

The amount of encapsulated CrAl_2_O_4_ particles was quantified using TGA in a temperature range of 50–800 °C. Figure 6 shows the TGA thermograms of P­(MMA-BA) microcapsules containing MPS-CrAl_2_O_4_ or o-CrAl_2_O_4_, prepared at MMA: BA ratios of 60:40 and 70:30. All samples exhibit a major weight loss associated with thermal degradation of the polymer matrix, followed by a stable residue at high temperatures corresponding to the inorganic CrAl_2_O_4_ phase. Based on the residual mass, the CrAl_2_O_4_ loadings were determined to be 12.0 and 9.2 wt % for MPS-CrAl_2_O_4_ microcapsules at MMA/BA ratios of 60:40 and 70:30, respectively. These results confirm the successful incorporation of CrAl_2_O_4_ in all formulations. The higher loading observed for the MPS-modified system at 60:40 suggests enhanced polymer–particle affinity due to surface functionalization. This observation correlated strongly with high %EE, 94% and 86% for MMA: BA of 60:40 and 70:30, respectively.

**Figure 6:**
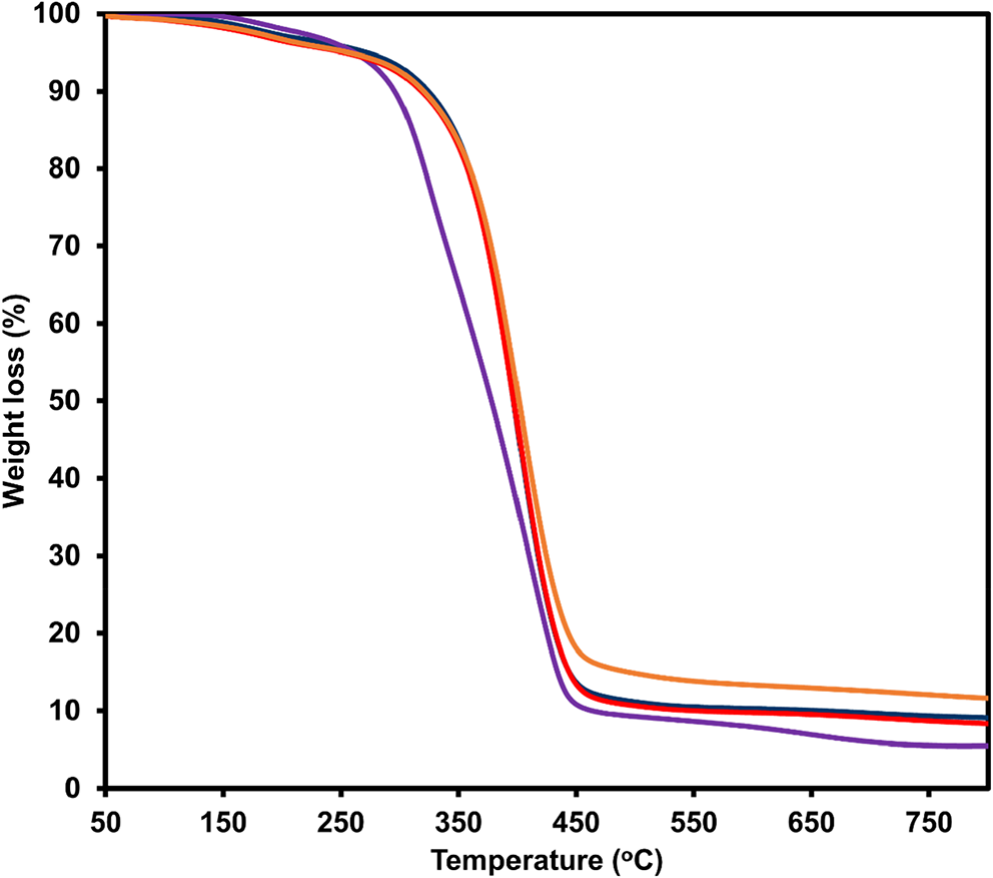
TGA thermograms of P­(MMA-BA) microcapsules encapsulating MPS-CrAl_2_O_4_ (orange and dark blue line) and o-CrAl_2_O_4_ particles (red and purple line) using various ratios of MMA/BA (%w/w): 60:40 (orange and red line) and 70:30 (dark blue and purple line).

When Tg was measured (Figure S4), the polymer microcapsules had significantly lower Tg values (−8.1 and −3.8 °C) than the polymer particles (3.3 and 8.6 °C) at ratios of 60:40 and 70:30, respectively. It is most likely because MPS modified on the surface of CrAl_2_O_4_ particles can be polymerized with MMA and BA monomers,^66^ allowing the CrAl_2_O_4_ particles to become strongly fixed in polymer microcapsules and function as a plasticizer, resulting in a decrease in the polymer′s Tg. This phenomenon promotes the uniform distribution and retention of CrAl_2_O_4_ particles in monomer droplets/polymer particles during polymerization. To clarify this hypothesis, CrAl_2_O_4_ particles modified with oleic acid (o-CrAl_2_O_4_), a small surfactant molecule, were compared. The green suspensions of polymer microcapsules encapsulated o-CrAl_2_O_4_ were observed, as shown in Figure 5(c,d). The well-distributed spherical monomer droplets (Figure 5c′,d′) and polymer microcapsules (Figure 5c″,d″) were also seen, as with MPS-CrAl_2_O_4_ particles. However, the loading of o-CrAl_2_O_4_ (9.0 and 8.5 wt %) in the microcapsules was lower than that of microcapsules encapsulating MPS-CrAl_2_O_4_, indicating improved encapsulation efficiency after MPS surface modification. Moreover, similar Tg values (3.1 and 8.4 °C) to those of the polymer particles were obtained.

Although rigid inorganic fillers generally increase the Tg by restricting polymer chain mobility, the present system is predominantly governed by the polymer–particle interfacial effects rather than the intrinsic rigidity of the CrAl_2_O_4_ particles themselves.^77^ In the case of MPS-modified CrAl_2_O_4_, the methacrylate groups of MPS can participate in the polymerization process, resulting in polymer chains being chemically grafted onto the particle surface. This chemical coupling generates a heterogeneous polymer–particle interfacial region with inefficient chain packing and increased local free volume, which counteracts the rigid filler effect and results in a reduced or unchanged overall Tg.^78,79^ In contrast, oleic-acid-modified CrAl_2_O_4_ primarily enhances particle dispersion through physical adsorption and steric stabilization without involvement in the polymerization reaction. As a result, the polymer network structure and chain mobility are largely retained, and the Tg remains close to that of the pristine polymer particles.^80^

Although stable suspensions with high %EE microcapsules were produced, they solidified into a glue-like state when kept at room temperature for a while. This is due to their low Tg, which causes microcapsule aggregation. As a result, the introduction of a cross-linked monomer to increase the stiffness of the microcapsule shell is being investigated further using MMA/BA at 60:40 due to its high %EE.

#### 3.3.2 Effect of Cross-Linked Comonomer

EGDMA was introduced as a cross-linking monomer to reinforce the polymer network. The presence of EGDMA increases network connectivity, which in turn improves the dimensional stability, mechanical strength, and thermal resistance of the polymer structure.^81,82^ In practical terms, this helps the microcapsules to maintain their structural integrity during processing and application. Various amounts of EGDMA cross-linked comonomer were added to increase the strength and Tg of the polymer microcapsules. At all ratios, spherical P­(MMA-BA-EGDMA)/MPS-CrAl_2_O_4_ microcapsules with relatively smooth surfaces were produced (Figure 7). Without EGDMA, aggregated microcapsules were clearly seen (Figure 7a), while they were well distributed with the addition of EGDMA (Figure 7b–d). Then, the addition of EGDMA effectively enhances the stability of the microcapsules, as expected. The incorporation of CrAl_2_O_4_ particles into the P­(MMA-BA-EGDMA) matrix was confirmed by EDS analysis. Figure 7 presents the EDS elemental mapping of the P­(MMA-BA-EGDMA)/MPS-CrAl_2_O_4_ microcapsules. A detailed examination of these maps clearly reveals the successful incorporation and uniform distribution of Cr (green spots), Al (red spots), and O (yellow spots) throughout the P­(MMA-BA-EGDMA) particles.

**Figure 7:**
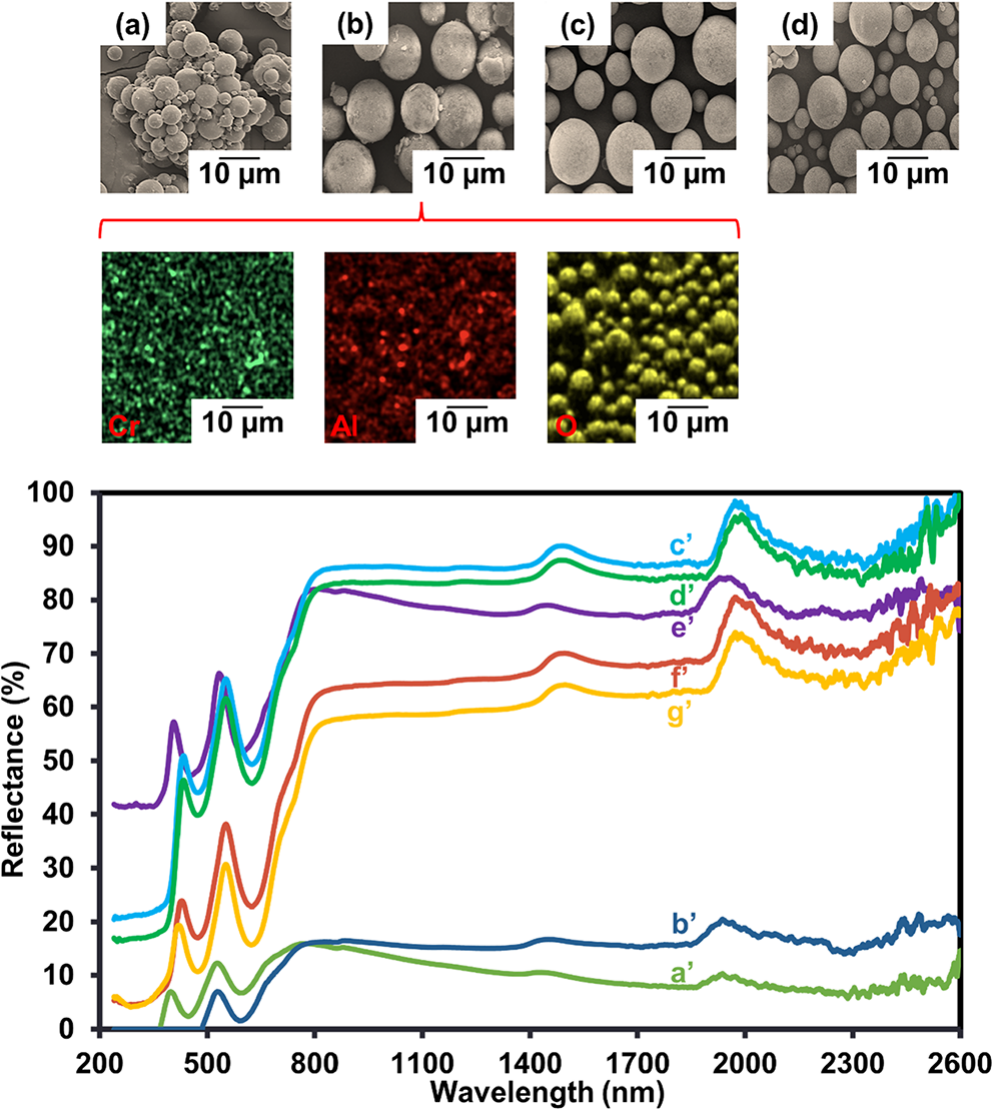
SEM-EDS micrographs of P­(MMA-BA-EGDMA)/MPS-CrAl_2_O_4_ microcapsules at various ratios of MMA: BA: EGDMA (%w/w): 60:40:0 (a); 50:40:10 (b); 40:40:20 (c) and 30:40:30 (d); and EDS analysis mapping using MMA: BA: EGDMA (%w/w) at 50:40:10. Reflective efficiency of bare glass (a′) and coated glass with P­(MMA-BA-EGDMA) particles (b′), CrAl_2_O_4_ particles (c′), and P­(MMA-BA-EGDMA)/MPS-CrAl_2_O_4_ microcapsules at various ratios of MMA: BA: EGDMA (%w/w): 60:40:0 (d′); 50:40:10 (e′); 40:40:20 (f′); and 30:40:30 (g′).

The addition of EGDMA did not influence the encapsulation efficacy of MPS-CrAl_2_O_4_, as %EE remained high at 95–98%. Tg values of P­(MMA-BA-EGDMA) at MMA: BA: EGDMA ratios of 60:40:0, 50:40:10, 40:40:20, and 30:40:30, respectively, were still lower than room temperature (−8.1, 12.6, 19.2, and 25.5 °C), which advantaged self-coating ability on the substrate.

Various P­(MMA-BA-EGDMA)/MPS-CrAl_2_O_4_ microcapsules were directly coated onto glass substrates without an external binder to assess the coating ability. The self-coating performance of the polymer microcapsules on the substrate is due to the superior film-forming characteristic of PBA.^24,25^ The polymer microcapsule films were evenly dispersed with MPS-CrAl_2_O_4_ particles after water evaporation. This finding demonstrated that P­(MMA-BA-EGDMA)/MPS-CrAl_2_O_4_ microcapsules displayed high colloidal stability and exhibited good dispersion in the medium throughout the coating process. However, the percentage add-ons of the polymer microcapsules reduced with increasing EGDMA concentration (97, 95, 69, and 52% for MMA/BA/EGDMA of 60:40:0, 50:40:10, 40:40:20, and 30:40:30, respectively). This is most likely a result of the polymer microcapsules’ diminished ability to form films due to an increase in EGDMA corresponding with their Tg. It is consistent with the previous report^25^ that self-coating performance decreased as the amount of cross-linked monomers increased.

Solar reflectance is crucial for cooling materials because it offers a theoretical assessment of the effectiveness of the cooling process.^25^ Then, the prepared P­(MMA-BA-EGDMA)/MPS-CrAl_2_O_4_ microcapsules’ ability to reflect NIR was investigated, and their performance was compared to that of P­(MMA-BA-EGDMA) and pristine CrAl_2_O_4_ particles. Polymer microcapsules with 0.05 g of CrAl_2_O_4_, equivalent to pristine CrAl_2_O_4_ particles, were used to cover a 6.25 cm^2^ glass substrate.

According to Figure 7a′, the bare glass constantly reflected low NIR light. Low NIR reflection performance along the UV–vis-NIR region was a bit enhanced and steadily maintained when the glass was coated with P­(MMA-BA-EGDMA) particles (Figure 7b′). Compared to pristine CrAl_2_O_4_, the NIR reflectance grew steadily, reaching a maximum of about 90% in the UV–vis-NIR region (Figure 7c′), indicating its excellent NIR reflection efficiency. However, coating with P­(MMA-BA-EGDMA) microcapsules lowered the NIR reflectance (Figure 7 d′–g′) even though they remained higher than 55% and reached close to about 85%. The NIR reflectance decreased with increased EGDMA content, probably due to the shielding of the cross-linking network of PEGDMA in the microcapsule shell.^25^ These findings suggest that the polymer matrix affects the scattered CrAl_2_O_4_ particles’ ability to reflect NIR light to some extent.

The irradiance-weighted average reflectance (R) values in the range of 240–2,600 nm for all samples were computed by eq 1, and the results are shown in Table 4 to compare the NIR reflectance quantitatively. The findings are consistent with solar reflectance in Figure 7 a′–g′. The *R* values of polymer microcapsule-coated glasses were significantly higher than those of bare glass. P­(MMA-BA-EGDMA)/MPS-CrAl_2_O_4_ microcapsules significantly raised the reflectance values, demonstrating the effectiveness of the encapsulated CrAl_2_O_4_ particles’ ability to reflect light, as indicated in Table 4. Using P­(MMA-BA-EGDMA)/MPS-CrAl_2_O_4_ microcapsules at a 60:40:0 ratio provided the highest value of 85.10%, which was close to that of the pristine CrAl_2_O_4_ (89.70%) because of its nondense PEGDMA network structure. However, they were unstable when stored for a long time. Thus, a 50:40:10 ratio was selected for further study to improve the reflection ability.

**Table 4:** Irradiance-Weighted Average Reflectance Values

sample	%*R*_(240–2,600 nm)_
glass substrate	9.26
P(MMA-BA-EGDMA) particles	13.69
CrAl_2_O_4_ particles	89.70
P(MMA-BA-EGDMA) microcapsules	
60:40:0	85.10
50:40:10	73.47
40:40:20	61.35
30:40:30	55.19

#### 3.3.3 Percentage of MPS-CrAl_2_O_4_ Particles

To maximize the efficiency of reflecting NIR light, several concentrations of CrAl_2_O_4_ particles were investigated. Polymer microcapsules were smoothly prepared under all conditions. The highly colloidal stable spherical microcapsules were formed with good distribution even at the highest CrAl_2_O_4_ loading up to 40 wt %. However, the presence of CrAl_2_O_4_ particles on the microcapsule surface was observed, as illustrated in Figure 8d–d′. The encapsulated contents of MPS-CrAl_2_O_4_ particles in the P­(MMA-BA-EGDMA) microcapsules were quantified by TGA, as shown in Figure S5. The pristine P­(MMA-BA-EGDMA) particles underwent almost complete thermal degradation, leaving negligible residue above 600 °C. In contrast, the residual mass increased systematically with increasing MPS-CrAl_2_O_4_ loading, indicating a progressively higher inorganic content within the microcapsules. Based on residual weights, the MPS-CrAl_2_O_4_ contents were estimated to be approximately 12.0, 18.3, 31.7, and 39.2 wt %. These results quantitatively confirm the effective and controllable incorporation of the inorganic phase into the polymer matrix, even at high loading levels. Nevertheless, the Tg values (12.6, 13.1, 11.9, and 12.4 °C for 10–40 wt % of MPS-CrAl_2_O_4_; Figure S6) were constant, indicating that the amount of CrAl_2_O_4_ particles did not affect the thermal properties of the polymer microcapsules.

**Figure 8:**
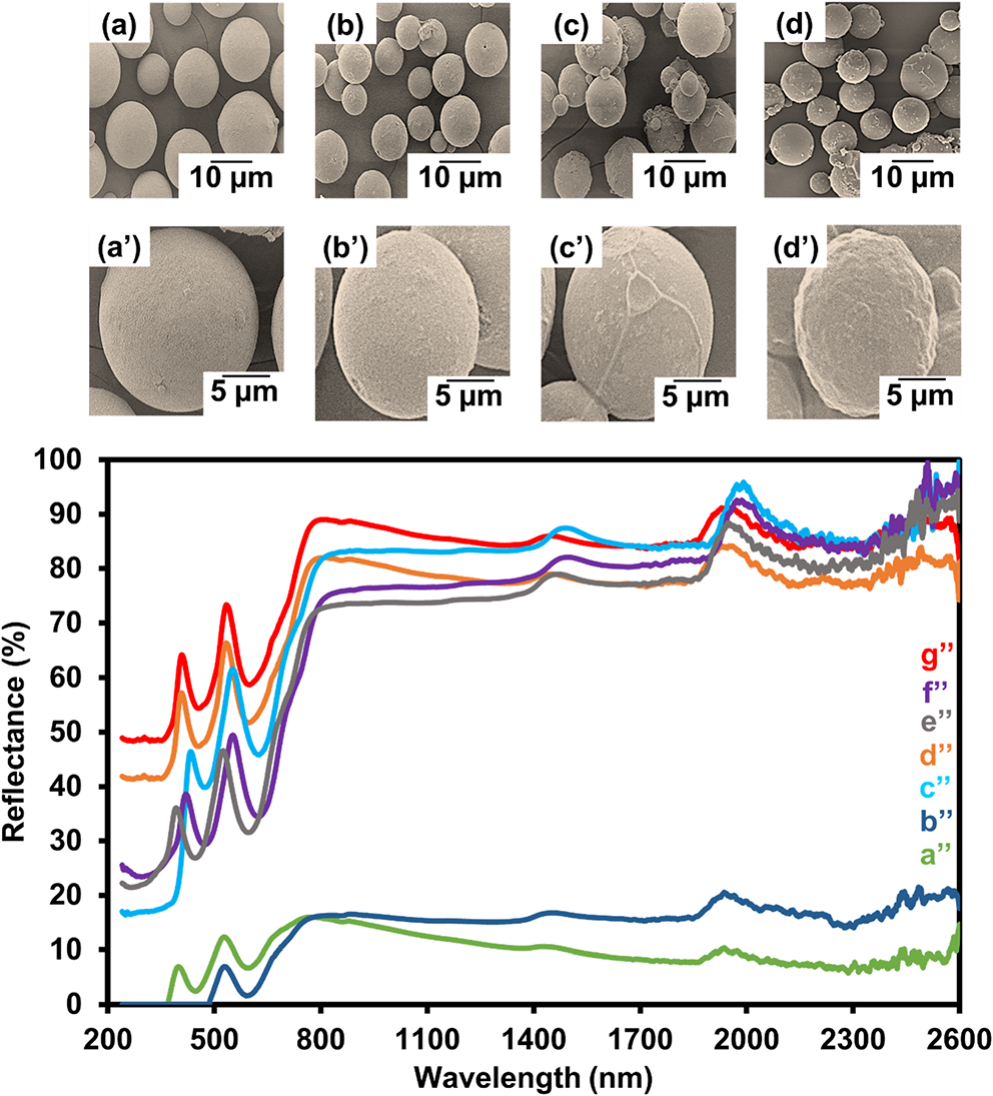
SEM micrographs of P­(MMA-BA-EGDMA)/MPS-CrAl_2_O_4_ microcapsules using various amounts of MPS-CrAl_2_O_4_ (wt %): 10 (a, a′); 20 (b, b′); 30 (c, c′); and 40 (d, d′) and reflective efficiency of bare glass (a″) and coated glass with P­(MMA-BA-EGDMA) particles (b″), CrAl_2_O_4_ particles (c″) and P­(MMA-BA-EGDMA)/MPS-CrAl_2_O_4_ microcapsules at various amounts of MPS-CrAl_2_O_4_ (wt %): 10 (d″); 20 (e″); 30 (f″); and 40 (g″).

### 3.4 Infrared Ray Reflection and Temperature Control Efficiency

#### 3.4.1 Infrared Ray Reflection

To evaluate their performances, the produced microcapsules’ NIR reflection and temperature control efficiencies were compared to pristine CrAl_2_O_4_ particles and bare glass. Without an external binder, P­(MMA-BA-EGDMA)/MPS-CrAl_2_O_4_ microcapsules were well adhered onto the glass substrate. The percentage add-ons of the P­(MMA-BA-EGDMA)/MPS-CrAl_2_O_4_ microcapsules on the substrates were remarkably consistent, at 95, 93, 96, and 92% for MPS-CrAl_2_O_4_ 10–40 wt %. Increasing the amount of MPS-CrAl_2_O_4_ has no effect on the percentage add-on because of the similar Tg of polymer microcapsules.

Figure 9 shows the SEM cross-sectional images of bare glass and glass coated with various particles. All samples exhibit a continuous and compact coating layer uniformly deposited on the glass surface. The thicknesses of the coatings, measured from multiple cross-sectional regions (Figure 9 b–f), are found to be approximately 18–20 μm, indicating consistent film formation across all formulations. The incorporation of MPS-CrAl_2_O_4_ particles does not significantly alter the overall coating thickness, as observed from the interface between the polymer matrix and the substrate. This uniform thickness ensures that the measured reflective efficiency of each coating can be directly correlated to the CrAl_2_O_4_ content rather than geometric differences in film thickness. Therefore, the SEM analysis confirms that the prepared coatings possess well-defined and uniform thicknesses (∼18–20 μm), suitable for consistent evaluation of NIR reflectivity and optical performance.

**Figure 9:**
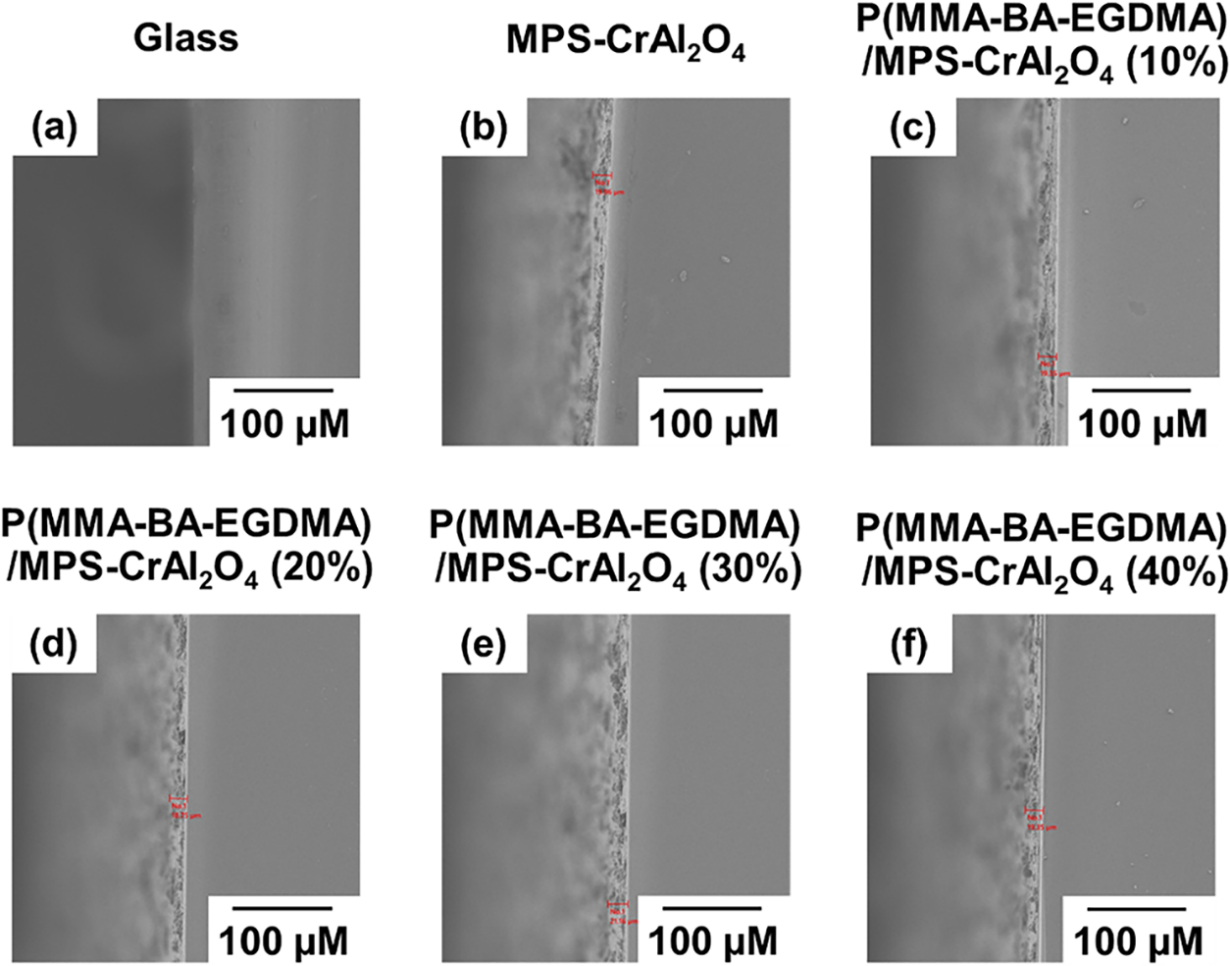
SEM images of bare glass (a) and glass coated with MPS-CrAl_2_O_4_ (b) and P­(MMA-BA-EGDMA)/MPS-CrAl_2_O_4_ microcapsules with various amounts of MPS-CrAl_2_O_4_ (wt %): 10 (c); 20 (d); 30 (e) and 40 (f).

Moreover, photographs of the glass substrates before and after coating were taken by using a digital camera. The uncoated glass exhibits high optical transparency, whereas the coated glass samples show a slightly cloudy appearance after being coated. This mild cloudiness becomes more pronounced with increasing CrAl_2_O_4_ content (12–39.2 wt %), which is consistent with the presence of inorganic particles dispersed within the polymer coating layer. The visible-light transmittance of the samples was further evaluated using UV–visible spectrophotometry. As shown in Figure S7, the bare glass displays the highest transmittance across the entire visible wavelength range. After coating, all samples exhibit only a slight reduction in transmittance of approximately 2–3%, while the overall spectral profiles remain largely unchanged. This decrease in transmittance is mainly attributed to light scattering induced by the polymer matrix and the dispersed CrAl_2_O_4_ particles rather than to significant absorption in the visible region. Importantly, even at the highest CrAl_2_O_4_ loading, the coated glass maintains relatively high visible transparency. These results indicate that the coating causes only a minor loss of optical clarity while preserving sufficient transparency for practical applications.

Solar reflectance is measured to determine the effectiveness of the cooling process. The *R* values in the 240–2,600 nm range for all samples were determined using eq 1 to quantitatively compare the NIR reflectance, as shown in Table 5. All polymer microcapsules were used with the same amount of the encapsulated CrAl_2_O_4_ (based on 40% loading). As illustrated in Figure 8 and Table 5, the bare glass substrate (a″) exhibits very low NIR reflectance across the entire wavelength range (240–2,600 nm), due to its high transmittance and lack of reflective or scattering components. This baseline demonstrates the minimal inherent capability of the uncoated substrate to block or reflect solar radiation. When coated with P­(MMA-BA-EGDMA) polymer particles without any pigment (b″), the reflectance slightly increases, attributed to surface scattering from the polymer layer. However, the polymer itself has no significant NIR-reflective properties and predominantly absorbs NIR radiation,^17,25^ resulting in only marginal improvement over bare glass. This indicates that the polymer matrix alone cannot provide sufficient NIR shielding and may even reduce overall reflectance when present in large amounts. In contrast, pristine CrAl_2_O_4_ particles (c″) show the highest reflectance among all samples, due to their spinel structure and strong intrinsic NIR-reflective capability. When CrAl_2_O_4_ is encapsulated into polymer microcapsules at various loading levels (10–40 wt %, curves d-g), a clear trend emerges: reflectance improves with increasing CrAl_2_O_4_ content. At lower loadings (10–20 wt %, curves d″ and e″), more polymer is required to deliver the same amount of pigment, leading to thicker shells and complete encapsulation. This excessive polymer reduces the effectiveness of the CrAl_2_O_4_ by absorbing NIR radiation and limiting the exposure of reflective surfaces, thereby diminishing the effectiveness of the reflective material. At higher CrAl_2_O_4_ loadings (30–40 wt %, curves f″ and g″), the amount of polymer decreases, resulting in thinner shells and partial surface exposure of CrAl_2_O_4_. This configuration allows the particles more direct reflection of incident NIR radiation, similar to the pristine CrAl_2_O_4_ particles, thereby boosting the overall reflectance significantly.^10,83,84^ The 40 wt % sample (curve g″) achieves reflectance nearly identical to the pristine CrAl_2_O_4_ (curve c″), confirming that minimizing polymer shielding while maintaining structural stability is key to optimizing performance. The NIR reflection efficiency of the CrAl_2_O_4_-loaded polymer microcapsules was benchmarked against other reported NIR-reflective capsules/composites, as summarized in Table S2.^10,17,25,85–91^ Notably, the CrAl_2_O_4_-loaded polymer microcapsules exhibit the highest NIR reflectance among the compared materials. This outstanding NIR reflection highlights their superior light-reflection capability in the solar heat-dominant region, underscoring their strong potential for heat-reflective and passive cooling coating applications.

**Table 5:** Irradiance-Weighted Average Reflectance Values

sample	%*R*_(240–2,600 nm)_
glass substrate	9.26
P(MMA-BA-EGDMA) particles	13.69
CrAl_2_O_4_ particles	89.70
P(MMA-BA-EGDMA)/MPS-CrAl_2_O_4_ microcapsules	
10% MPS-CrAl_2_O_4_	73.47
20% MPS-CrAl_2_O_4_	75.13
30% MPS-CrAl_2_O_4_	78.56
40% MPS-CrAl_2_O_4_	86.22

#### 3.4.2 Temperature Control Efficiency

Since solar reflection provides only an indirect and theoretical estimation, temperature testing was conducted to verify the material’s practical cooling effectiveness.^44^ In this study, a temperature test was conducted to directly assess the temperature regulation efficiency of the prepared polymer microcapsules. The temperature control performance was expressed as the temperature difference (ΔT) between the upper surface of the substrate, which was directly irradiated by a lamp, and the lower surface. The results, illustrated in Figure 10, demonstrate the thermal regulation performance of glass substrates coated with various particles, including CrAl_2_O_4_, P­(MMA-BA-EGDMA)/MPS-CrAl_2_O_4,_ and P­(MMA-BA-EGDMA). The ΔT of the bare glass remained consistently at about 2 °C, mainly due to its high transmittance, allowing most of the light to pass through without significant absorption. In contrast, glass coated with P­(MMA-BA-EGDMA) particles exhibited a slight increase in ΔT to approximately 3 °C throughout the experiment, indicating limited thermal shielding performance. Outstandingly, glass coated with P­(MMA-BA-EGDMA)/MPS-CrAl_2_O_4_ microcapsules displayed a substantial temperature difference. The microcapsules with a 50:40:10 wt % ratio and 40 wt % CrAl_2_O_4_ particles show the largest ΔT, which is close to that of pristine CrAl_2_O_4_ particles at about 16–17 °C. The results are in good agreement with %R. This represents a temperature reduction approximately 8–9 times greater than that observed for uncoated glass, even under prolonged irradiation. The pronounced thermal insulation effect is attributed to the presence of CrAl_2_O_4_-encapsulated microcapsules, which effectively block the transmission of NIR radiation. As a result, the lower substrate temperature was significantly reduced compared to the irradiated upper surface. These findings indicate that high solar reflection contributes to improved cooling performance, which is in good agreement with the NIR reflectance values presented in Figure 8.

**Figure 10:**
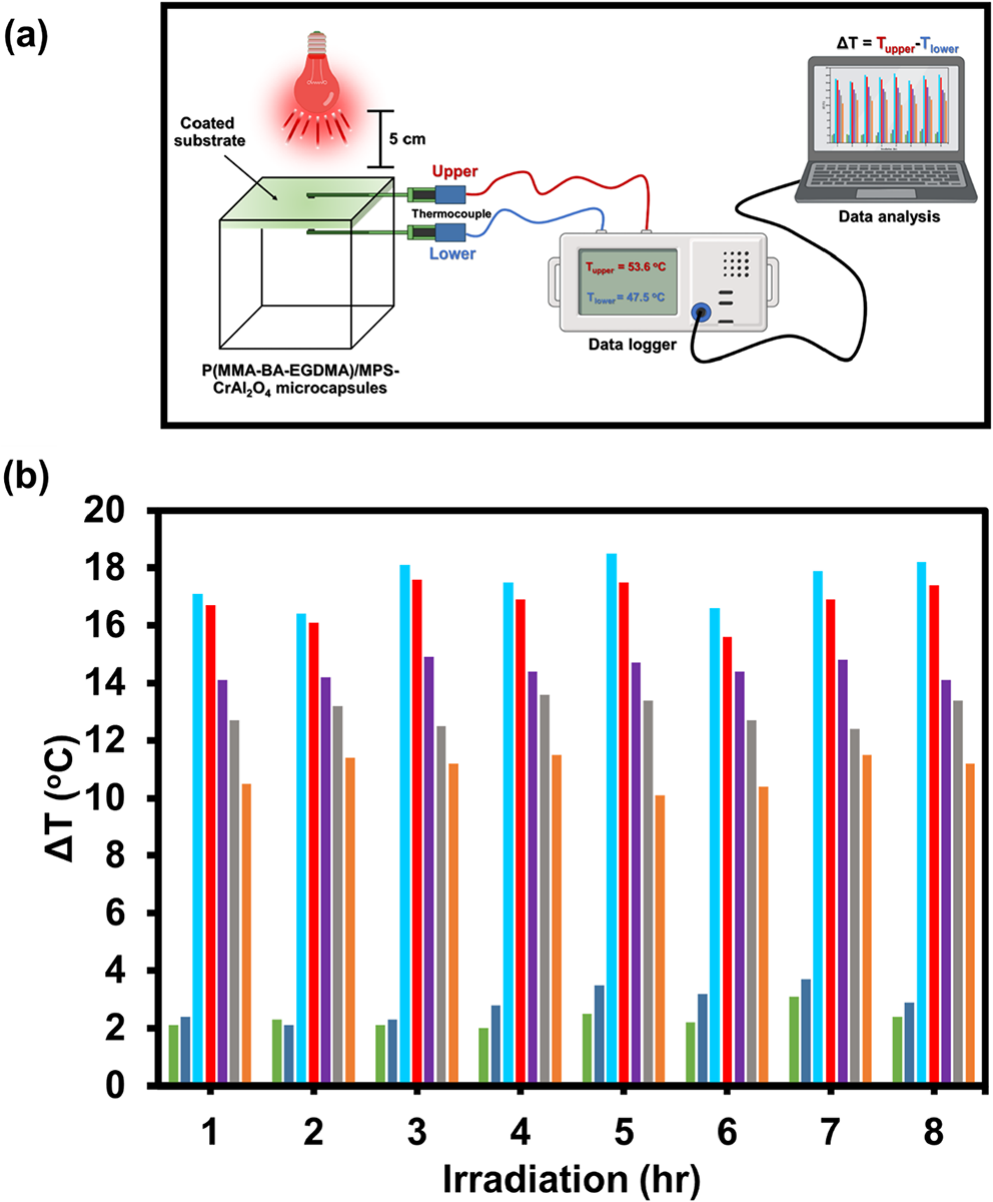
Schematic diagram of temperature differences measurement (a) and temperature differences (ΔT) (b) at various irradiation times of bare glass (green) and coated glass with P­(MMA-BA-EGDMA) particles (dark blue), CrAl_2_O_4_ particles (light blue), and P­(MMA-BA-EGDMA)/CrAl_2_O_4_ particles at various contents of MPS-CrAl_2_O_4_ (wt %): 10 (orange); 20 (gray); 30 (purple); and 40 (red).

### 3.5 Durability of Coated Glass Substrate

The durability and weatherability of the resulting coatings were preliminarily evaluated using accelerated UV and water exposure tests in accordance with ASTM D4329 and ASTM D870. As shown in Figure 11, a total of five sample formulations were investigated, and each formulation was tested in triplicate in the durability experiment. The results are presented as mean ± standard deviation (SD) and exhibited a gradual mass loss during cyclic UV irradiation and water immersion for up to 720 h. This behavior is mainly attributed to minor polymer degradation and surface erosion under combined UV and moisture stress, which is commonly reported for acrylic-based coatings under accelerated weathering conditions.^30,92,93^ Importantly, the incorporation of CrAl_2_O_4_ particles did not result in catastrophic degradation or delamination of the coatings. Microcapsules containing 10–30 wt % CrAl_2_O_4_ exhibited mass-loss trends comparable to, or slightly lower than, those of the neat polymer particles. This observation indicates that the inorganic spinel particles do not compromise coating integrity and may partially mitigate UV-induced degradation by reducing UV penetration and acting as physically stable fillers, consistent with previous reports on inorganic oxide-filled UV-resistant coatings.^54,94^ At higher loadings (40 wt %), a slightly increased mass loss was observed, which can be attributed to enhanced interfacial stress and increased water accessibility at high inorganic contents, rather than to failure of the self-coating mechanism itself.^95^ In this study, the absence of sudden mass loss or peeling during repeated UV-water cycles suggests that the coatings maintain sufficient adhesion and mechanical integrity under harsh environmental conditions, as mass stability under cyclic weathering is widely used as an indirect indicator of coating durability.^30,93,95^ Previous studies using ASTM D4329 and ASTM D870 have generally adopted these standards as procedural frameworks for accelerated weathering and water-immersion testing, respectively, while reporting degradation in terms of measurable changes such as mass variation, water absorption, or surface damage.^24,25,92,96–98^ In most cases, no universal numerical acceptance criterion is prescribed, and the practical assessment of degradation is defined according to the material system under investigation. In line with this, an acceptance criterion for degradation was established in this work based on mass retention after 720 h of testing. Specifically, samples retaining more than 80% of their initial mass were considered to exhibit acceptable stability under the applied conditions. These results demonstrate that the proposed self-coating system exhibits reasonable weatherability and resistance to UV and moisture exposure, supporting its potential for surface and interfacial applications.

**Figure 11:**
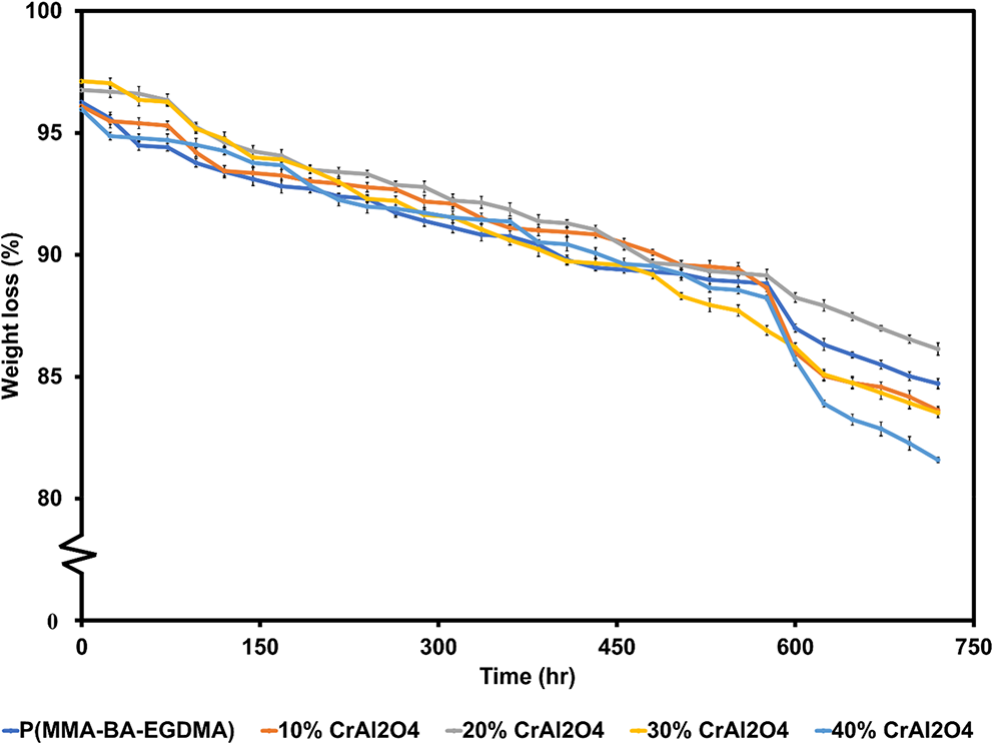
Durability and weatherability in terms of mass loss of the glass substrates coated with P­(MMA-BA-EGDMA)/MPS-CrAl_2_O_4_ microcapsules using various amounts of MPS-CrAl_2_O_4_ (wt %): 0 (blue line); 10 (orange line); 20 (gray line); 30 (yellow line); and 40 (sky blue line) [Data are presented as mean ± standard deviation (*n* = 3)].

## 4 Conclusions

In this work, spinel-structured CrAl_2_O_4_ particles were investigated as NIR-reflective materials and successfully incorporated into polymer microcapsules via UV-initiated microsuspension polymerization. The results showed that CrAl_2_O_4_ particles could be encapsulated within a polymer shell while maintaining the film-forming ability and dispersion stability. Among the studied formulations, the composition with MMA/BA/EGDMA = 50:40:10 and 40 wt % CrAl_2_O_4_ exhibited the highest reflectance, with values close to those of the raw pigment. In the model coating test used in this study, the microcapsule-coated glass showed a lower interior temperature than the uncoated glass, indicating the potential of the encapsulated pigment system to reduce heat transfer under the tested conditions. Overall, these findings demonstrate that CrAl_2_O_4_-containing polymer microcapsules are a promising approach for preparing NIR-reflective coating materials.

## Supplementary Material



## Data Availability

The data supporting the findings of this study will be made available upon request.

## Notes

NIR reflectance comparison of CrAl_2_O_4_ particles and microcapsules (Tables S1, S2); phase behavior and contact angle of MPS-modified CrAl_2_O_4_ (Figure S1); particle size distributions (Figure S2); DSC and TGA thermograms of polymer particles and microcapsules with varying compositions (Figures S3–S6); and optical images and transmittance spectra of coated substrates (Figure S7) (PDF)
