# Supplementary material for: Highly Efficient NIR-Reflective CrAl2O4‑Based Polymer Microcapsules for Advanced Thermal Management Coatings
Source: ACS Omega. 2026 May 15;11(21):31600–17. doi: 10.1021/acsomega.6c02354 (PMC13234644; doi:10.1021/acsomega.6c02354)
Supplement: Supplementary file 1 [file ao6c02354_si_001.pdf]

Supporting information

Highly Efficient NIR-Reflective $CrAl_2O_4$-Based Polymer Microcapsules for Advanced Thermal Management Coatings

Jittipat Omsinsombon[1], Amorn Chaiyasat[1,2], Chumphol Busabok[3] and Preeyaporn Chaiyasat[1, 2*]

[1]Department of Chemistry, Faculty of Science and Technology, Rajamangala University of Technology Thanyaburi, Pathum Thani, Thailand 12110

[2]Advanced Materials Design and Development (AMDD) Research Unit, Faculty of Science and Technology, Rajamangala University of Technology Thanyaburi, Pathum Thani, Thailand 12110

[3]Expert Centre of Innovative Materials, Thailand Institute of Scientific and Technological Research (TISTR), Khlong Luang, Pathum Thani, Thailand 12120

E-mail: p_chaiyasat@mail.rmutt.ac.th

Table S1. Comparing $CrAl_2O_4$ particles with other reflective materials

No.	Reflective materials	Investigated Wavelength	IR reflectance efficiency (%)	Reference
**1**	**$CrAl_2O_4$ particle**	**NIR solar (700-2,500 nm)**	**89.70**	**This work**
2	TiO_2	NIR solar	80.13	[10]
3	Lanthanum-strontium-copper silicates	NIR solar	65	[11]
4	$CoAl_2O_4$ spinel pigment	NIR solar	~60.2	[12]
5	TiO_2-$CoAl_2O_4$ composite pigment	NIR solar	>80.6	[46]
6	Mg^{2+} and Al^{3+} Co-Doped $CoCr_2O_4$ Inorganic Pigments	NIR solar	varies, often <90	[50]
7	(Ti)-doped Cr2O3 cool pigments	NIR solar	84–91 (pure ~84)	[45]
8	$Ca_2Mn_{0.85}Ti_{0.15}O_4$ (black pigment)	NIR solar	~66.2	[47]
9	Fe_2O_3@SiO_2 core–shell red pigment	NIR solar	62.7–65.3	[49]
10	Mn/Fe/Co/Ni-Dope $SrTiO_3$ perovskite pigments	NIR solar	45–77	[52]
11	$Y_{0.4}Bi_{0.6}P_{0.4}V_{0.6}O_4$ yellow pigment	NIR solar	~54.6	[51]

Table S2. Comparing $CrAl_2O_4$ microcapsules with other microcapsules

No.	Materials	IR reflectance efficiency (%)	Reference
**1**	**$CrAl_2O_4$-loaded polymer microcapsules**	**86.22 (NIR)**	**This work**
2	ZnO-PMMA hybrid coating cool-textile	50.00 (NIR)	[86]
3	$FeAl_2O_4$ composite polymer particle	67.22 (NIR)	[25]
4	Hollow fly ash bead/TiO_2 composite pigment	68 (NIR)	[87]
5	Hollow ZnO/SiO_2 (or Zn-silicate/SiO_2) coating pigment	65 (NIR)	[90]
6	Polymer-encapsulated TiO_2	80 (NIR)	[10]
7	Polymer-encapsulated colorful Al pigment	80.03 (NIR)	[17]
8	PMMA/M-ST composites	70.7 (NIR)	[88]

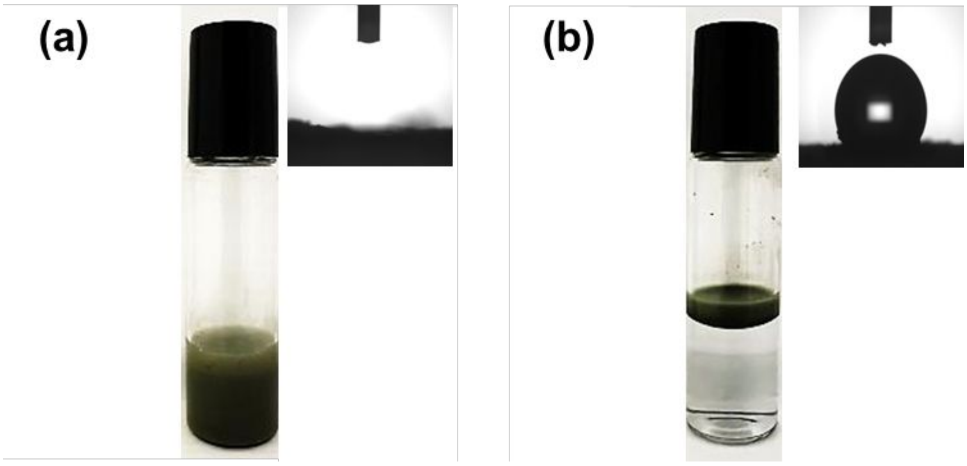

Figure S1. Binary phase photos and contact angle of partitioning study of MPS-$CrAl_2O_4$ particles with $CrAl_2O_4$: MPS ratios (%w/w): 100: 0 (a), and 50: 50 (b)

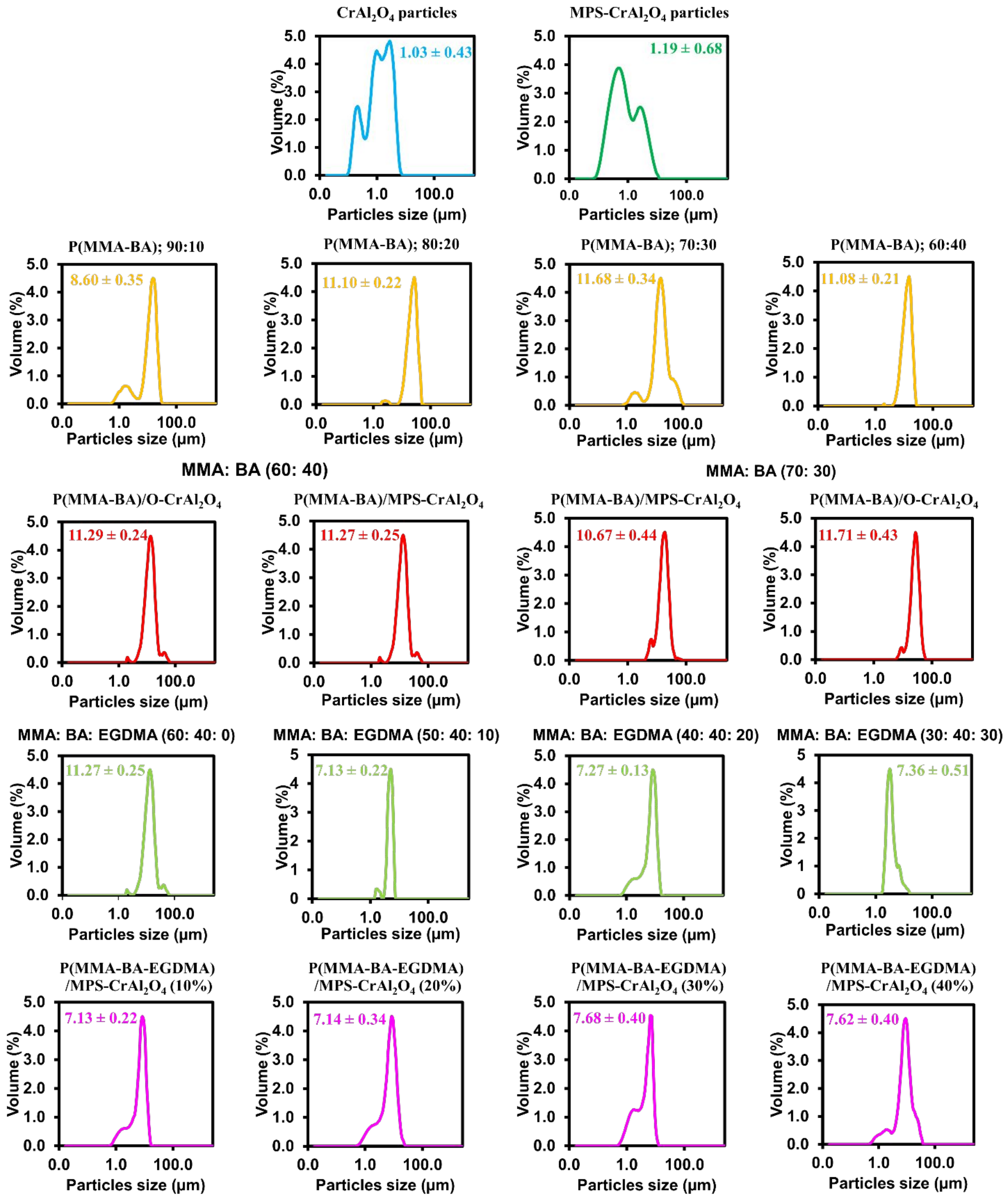

Figure S2. The particle size distribution histograms of P(MMA-BA), P(MMA-BA)/o-$CrAl_2O_4$, P(MMA-BA)/MPS-$CrAl_2O_4$ and P(MMA-BA-EGDMA)/MPS-$CrAl_2O_4$ microcapsules [Data are presented as mean ± standard deviation (n = 3)]

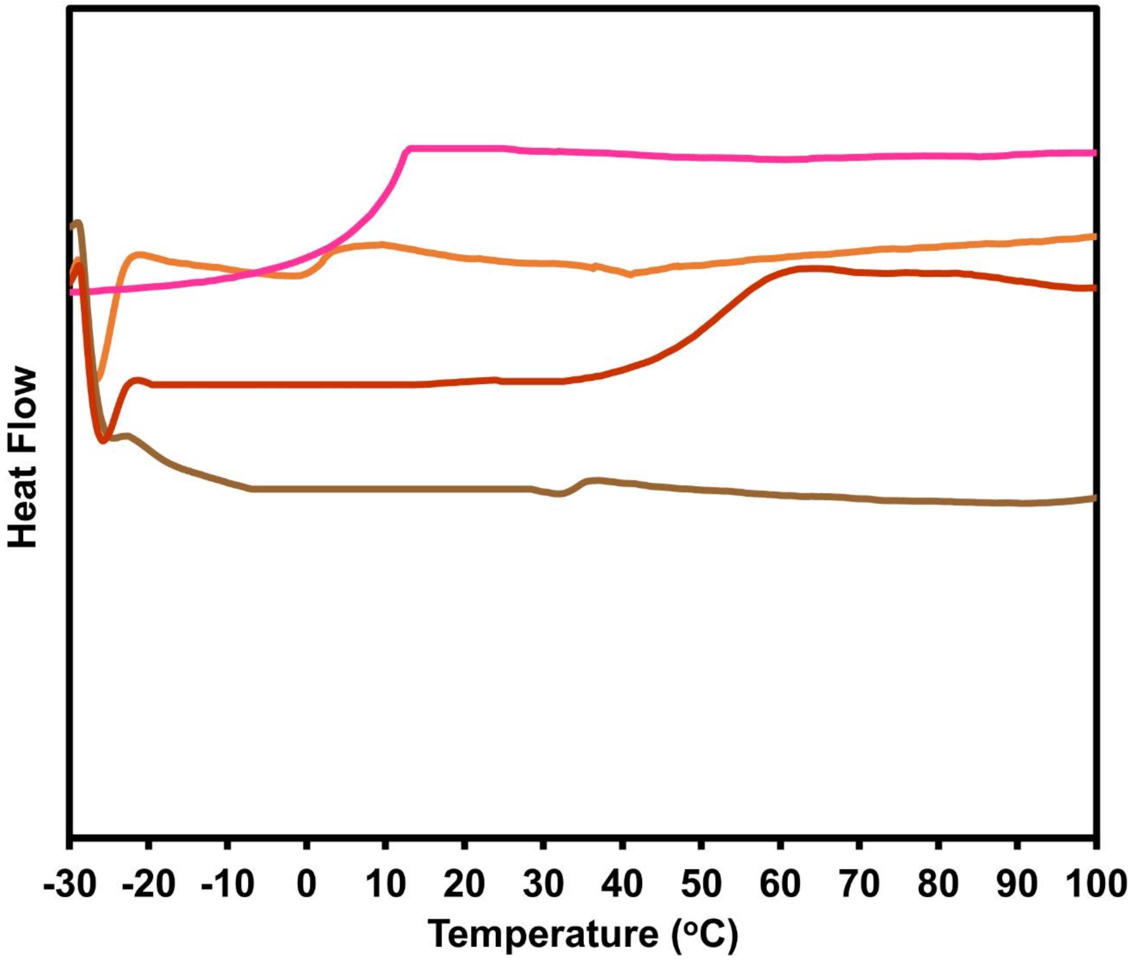

Figure S3. DSC thermograms of P(MMA-BA) particles using various ratios of MMA: BA (%w/w): 60: 40 (Orange line); 70: 30 (Pink line); 80: 20 (Brown line), and 90: 10 (Crimson line)

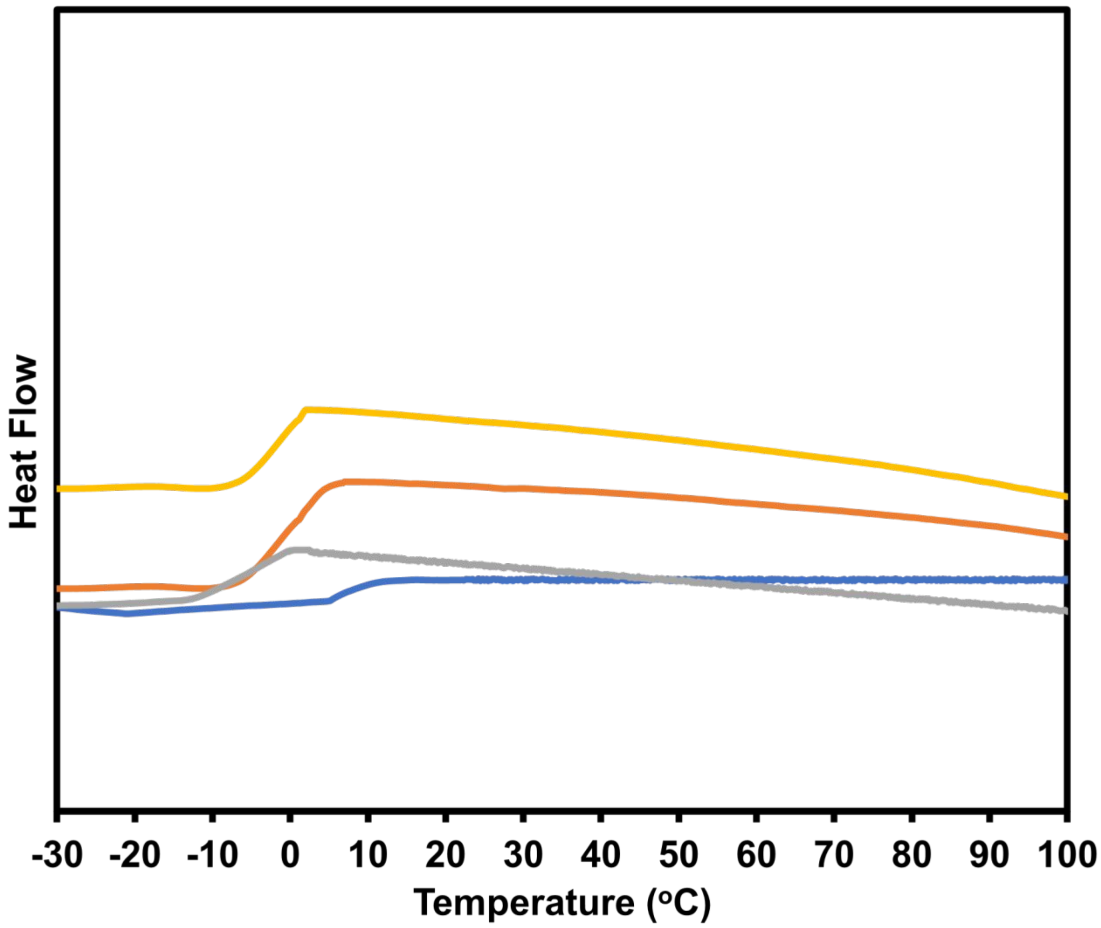

Figure S4. DSC thermograms of P(MMA-BA) microcapsules encapsulating MPS-$CrAl_2O_4$ (Gray and Yellow line) and o-$CrAl_2O_4$ particles (Orange and Blue line) using various ratios of MMA: BA (%w/w): 60: 40 (Gray and Orange line), and 70: 30 (Yellow and Blue line)

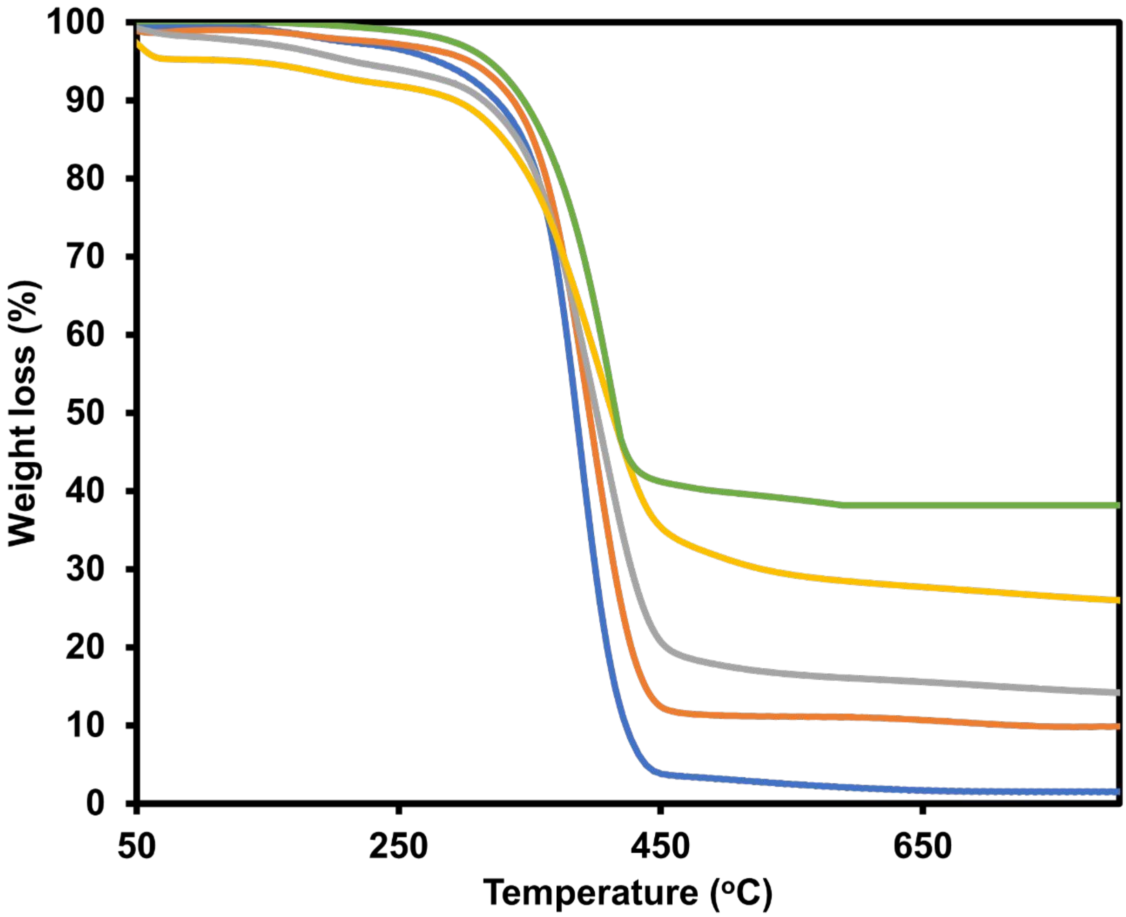

Figure S5. TGA thermograms of P(MMA-BA-EGDMA)/MPS-$CrAl_2O_4$ microcapsules using various amounts of MPS-$CrAl_2O_4$ (wt%): 0 (Blue line); 10 (Orange line); 20 (Grey line); 30 (Yellow line), and 40 (Green line)

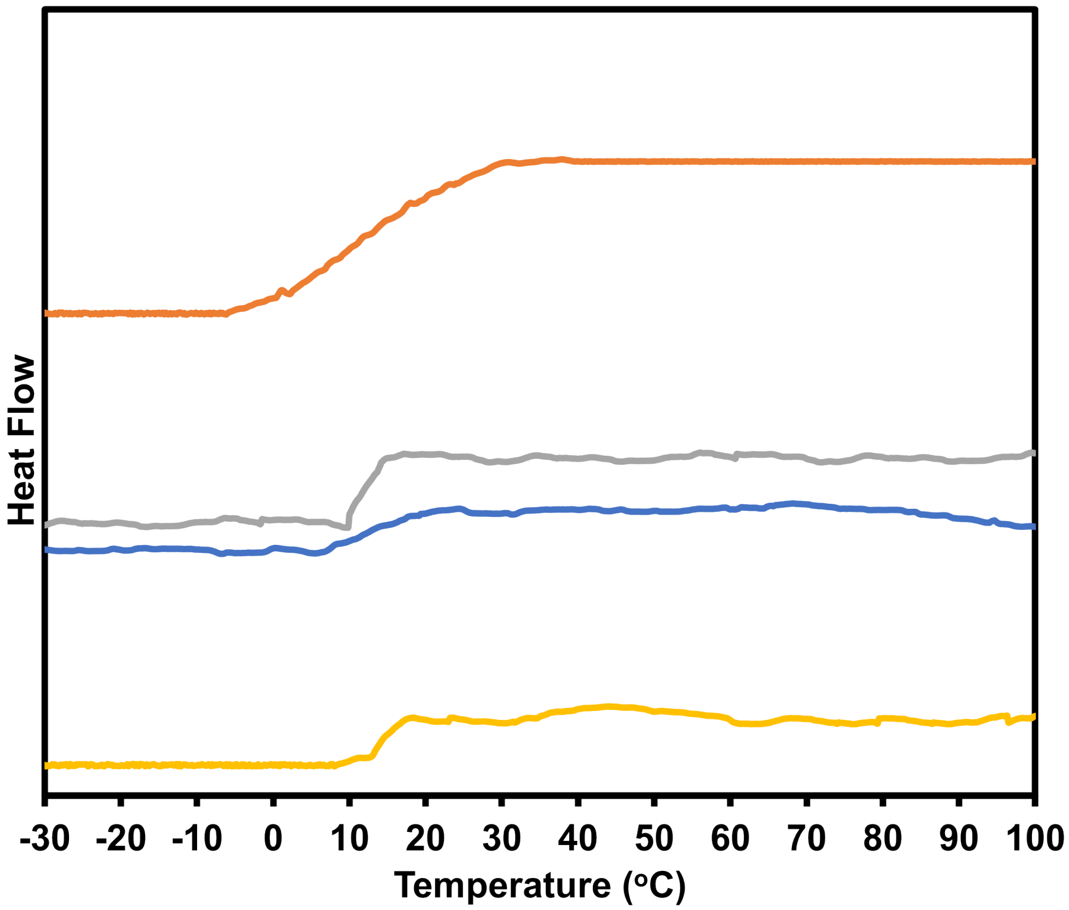

Figure S6. DSC thermograms of P(MMA-BA-EGDMA)/MPS-$CrAl_2O_4$ microcapsules using various amounts of MPS-$CrAl_2O_4$ (wt%): 10 (Yellow line); 20 (Blue line); 30 (Gray line), and 40 (Orange line)

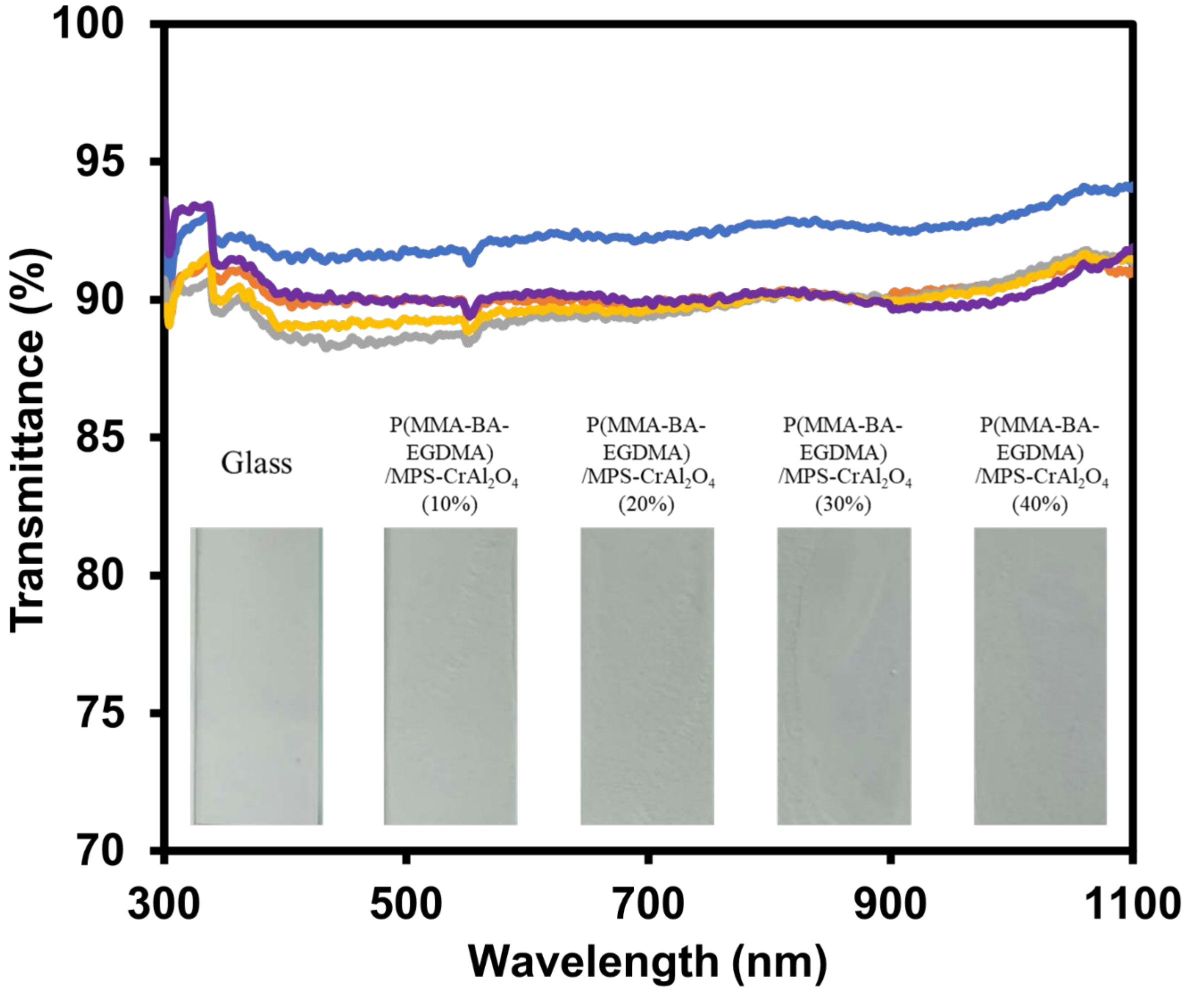

Figure S7. Photographs and transmittances (%) of the glass substrates before (Blue line) and after coating with P(MMA-BA-EGDMA)/MPS-$CrAl_2O_4$ microcapsules at various amounts of MPS-$CrAl_2O_4$ (wt%): 10 (purple line); 20 (Orange line); 30 (Yellow line), and 40 (Gray line)
